# Fluctuation of Global Gene Expression by Endogenous miRNA Response to the Introduction of an Exogenous miRNA

**DOI:** 10.3390/ijms140611171

**Published:** 2013-05-27

**Authors:** Yoshiro Nagata, Eigo Shimizu, Naoki Hibio, Kumiko Ui-Tei

**Affiliations:** 1 Department of Computational Biology, Graduate School of Frontier Sciences, University of Tokyo, 5-1-5 Kashiwanoha, Kashiwa-shi, Chiba-ken 277-8561, Japan; E-Mails: a5205075@gmail.com (Y.N.); lapcat.nh@gmail.com (N.H.); 2 Department of Biophysics and Biochemistry, Graduate School of Science, University of Tokyo, 7-3-1 Hongo, Bunkyo-ku, Tokyo 113-0033, Japan; E-Mail: eigo0shimizu@gmail.com

**Keywords:** exogenous microRNA, endogenous microRNA, microarray, seed-matched target

## Abstract

Most of the intracellular endogenous microRNAs (endo-miRNAs) are considered to be saturated in Argonaute (Ago) proteins in the RNA-induced silencing complexes (RISCs). When exogenous miRNAs (exo-miRNAs) are introduced into cells, endo-miRNAs in the RISC may be replaced with exo-miRNAs or exo-miRNAs, and endo-miRNAs might also compete for the position in the newly synthesized RISC with each other. This would lead to the fluctuation of global gene expression not only by repression of exo-miRNA target gene expression, but also by the increase of the endo-miRNA target gene expression. In the present study, we quantified the changes in the expression levels of target genes of exo-miRNA and endo-miRNA in the cells transfected with fifteen different exo-miRNAs by microarray experiments. Different exo-miRNAs increased ratios of expression levels of target genes of a given endo-miRNA to different extents, suggesting that the replacement efficiencies might differ according to the exo-miRNA types. However, the increased ratios in the expression levels of each endo-miRNA target genes by the transfection of any particular exo-miRNA were mostly equivalent, suggesting that the endo-miRNAs present in the RISC might be replaced with excessive exo-miRNAs at similar levels, probably because they exist in single-stranded forms in the RISC.

## 1. Introduction

MicroRNAs (miRNAs) are an abundant class of non-coding RNAs, about ~22 nucleotides long, that are key posttranscriptional regulators of gene expression in various organisms, including animals, plants and protozoa [1–3]. Most endogenous miRNAs (endo-miRNAs) actively silence target genes mainly by contiguous and perfect Watson-Crick base-pairing between the miRNA 5′-proximal seed region (positions 2–8) and its complementary sequences in 3′ untranslated regions (3′ UTRs) of target genes [4–6]. However, transfection of the small interfering RNA (siRNA)/miRNA expression construct into cells relieves repression of the target genes of endo-miRNAs dose-dependently at low concentrations and reaches the saturation level at high concentration [7–10].

These interfering effects are shown to be caused by competition for RNA silencing components. One of the key components is the nuclear karyopherin Exportin-5 [11–13], which binds to siRNA/miRNA precursors to transport them from the nucleus to the cytoplasm in the presence of Ran-GTP. The overloading of Exportin-5 by the excessive production of hairpin-structured siRNAs/miRNAs transcribed from their expression constructs could result in a decrease of cellular miRNA function [7,14]. However, the silencing activities of siRNA/miRNA duplexes are not affected by Exportin-5, since they do not need to be transported from the nucleus to the cytoplasm for their function [8,10,11]. Nonetheless, siRNA/miRNA duplexes have been shown to cause upregulation of their non-target genes by competition with endo-miRNAs. They compete for another saturable component of RNA silencing machinery, the RNA-induced silencing complex (RISC) [8,10,15]. The displacement of endo-miRNAs from the RISC by the introduction of synthetic siRNAs/miRNA duplexes are shown to be observed as the increase of endo-miRNA target gene expression [10].

RISC is the cytoplasmic effector machine of the miRNA silencing pathway. RISC assembly is mediated by the RISC loading complex, which is a multi-protein complex composed of the core protein, Argonaute (Ago), the RNase Dicer and the double-stranded RNA-binding protein, TRBP (TAR RNA binding protein) [16–19]. Initially, miRNA duplexes and siRNAs are loaded into Ago protein contained in the RISC loading complex [20,21]. To form the active RISC that performs gene silencing, the small RNA duplex needs to be separated or unwound into the single stranded form guiding it to its target mRNAs, within Ago protein. Then Dicer and its interactor TRBP dissociate from the RISC. Generally, miRNA forms an imperfect duplex composed of a miRNA strand and an opposite-strand miRNA. Evolutionary pressure has selected one particular strand of the duplex as the main regulator, which is preferentially loaded onto RISC, with the opposite strand being less functional [22,23]. The strand choice is considered to be not random and is partly determined by the intrinsic sequence and/or mismatched base-pairing of the miRNA duplex. The major determinants of RISC loading have been shown to be the thermodynamic properties: the strand with the less stable 5′ end is more often loaded onto active RISC [24–26]. Furthermore, central mismatches of miRNAs have also been shown to promote RISC loading [27]. In the cells, RISC is considered to be saturated with endo-miRNAs. Because these experiments were carried out by the transfection of exogenous miRNAs (exo-miRNAs), exo-miRNAs are anticipated to exclude the endo-miRNAs from RISC or compete with endo-miRNAs for RISC with each other.

In this study, to investigate the mechanism to perturb the endo-miRNA function caused by the introduction of exo-miRNA, we performed microarray profiling to quantify changes in the expression levels of endo-miRNA target genes following the transfection of fifteen different synthetic exo-miRNA duplexes. Such competition among cellular miRNAs is considered to have a role in normal biological and disease-related cellular processes. The exo-miRNAs, in addition to silencing their own target genes, clearly increased the expression of endo-miRNA target genes. The increased levels of endo-miRNA target gene expression were varied according to the types of the transfected exo-miRNA duplexes, suggesting each exo-miRNA duplex is presumed to have distinct characteristics, such as structures and sequences, which affect its incorporation into the RISC. However, the increased levels were mostly equivalent according to the types of endo-miRNAs after transfection of any particular exo-miRNA duplex. These results might propose the possibility that the endo-miRNAs present in the RISC in single-stranded form, but not in duplex form, nor in target-pairing form, were replaced with exo-miRNA duplexes, because double-stranded endo-miRNAs with different characteristics might not be replaced with exo-miRNAs in the similar levels. A part of exo-miRNAs transfected might compete with endo-miRNAs for newly synthesized Ago proteins, but such effects might be negligible, because the transfected exo-miRNAs are sufficiently abundant. Furthermore, the endo-miRNA target genes with a large number of target sites in their 3′ UTRs are revealed to be strongly repressed by endo-miRNAs in normal conditions. So, the genes with short 3′ UTRs, which have a small number of miRNA target sites, were weakly repressed by endo-miRNAs in the normal condition, then efficiently repressed when exo-miRNAs were transfected.

## 2. Results and Discussion

### 2.1. Microarray Profiling of the Reduced Expression of Exo-miRNA Target Genes

**Figure 1:**
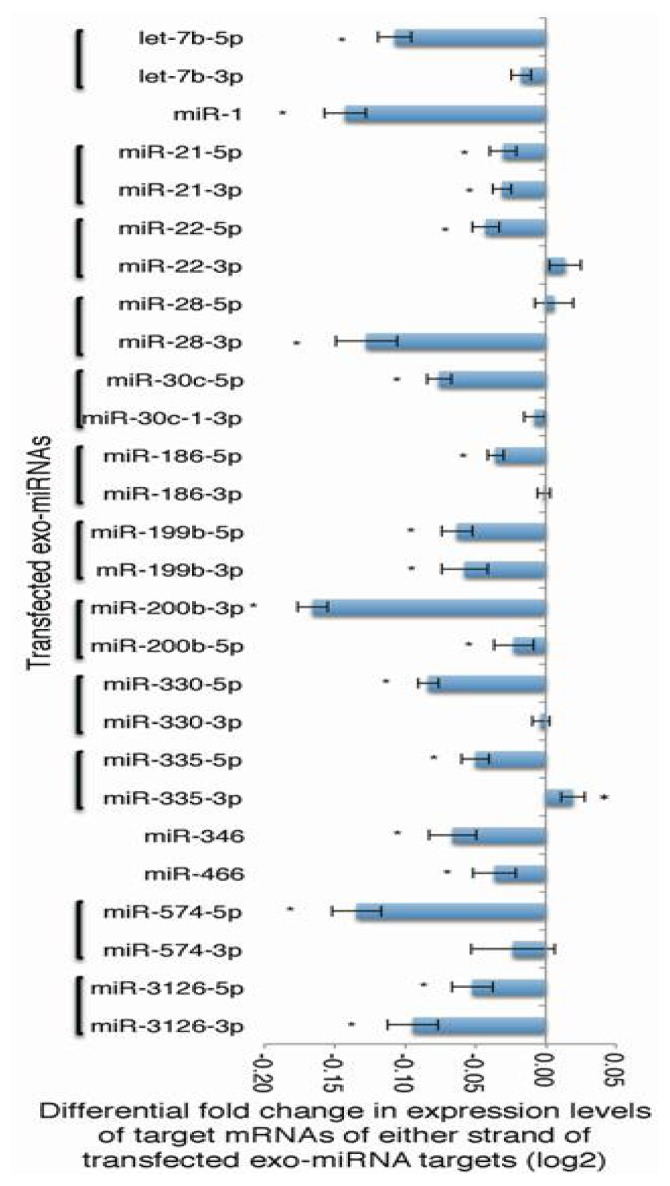
Microarray analysis of exo-miRNA target gene expression. HeLa cells transfected with each of exo-miRNA duplexes were subjected to microarray profiling. Differential fold changes in gene expression caused by the transfection of either strand of fifteen different exo-miRNAs. The results of the opposite strands of miR-1, miR-346 and miR-466 were not shown, because these miRNAs were not registered in miRBase. The MA plots and cumulative distribution patterns are shown in Figure S1.

To quantify global changes in the expression levels of endo-miRNA target genes resulting from the transfection of various exo-miRNA duplexes, microarray experiments were carried out using fifteen different exo-miRNA duplexes (let-7b, miR-1, miR-21, miR-22, miR-28, miR-30c-1, miR-186, miR-199b, miR-200b, miR-330, miR-335, miR-346, miR-466, miR-574 and miR-3126), which were chemically synthesized to form the duplex structures the same as those shown in miRBase [28]. At first, 24 h following the transfection of each exo-miRNA duplexes into human HeLa cells, the effects on own target genes of either of both miRNA strand were examined. We chose this time point, because it is reported that RNA silencing is generally maximal ~24 h post-transfection and that protein silencing varies depending on the target, but is generally maximal ~48–72 h post-transfection [29]. Thus, the result at 24 h post-transfection may largely reflect the direct effects without downstream effects of exo-miRNAs, since major downstream effects should be observed after 48 h post-transfection. It has been reported that miRNA target genes mainly contain sequences complementary to miRNA seed regions at positions 2–8 from the 5′ terminus [4–6], then the expression patterns of these genes were analyzed. The expression patterns of each miRNA target genes containing seed-complementary sequences in HeLa cells transfected with fifteen exo-miRNAs are shown as MA (M = intensity ratio, A = average intensity) plots (Figure S1A-1~O-1 and S1A-3~O-3) and cumulative distributions (Figure S1A-2~O-2 and S1A-4~O-4). The changes in expression were calculated as the difference values subtracting the average fold changes (log_2_) for exo-miRNA seed-matched target genes (blue line in Figure S1A-2~O-2 and S1A-4~O-4) from the average value for the genes without seed-matched sequence (black line in Figure S1A-2~O-2 and S1A-4~O-4). The differential fold change values were between 0.02 and −0.17, as summarized in Figure 1, indicating that all of the exo-miRNAs used in this study could decrease their own target genes at the different levels, due to the miRNA species. However, these decreases in exo-miRNA target gene expression, as a result of competition, with the corresponding endo-miRNAs might be underestimated in this experiment.

### 2.2. Microarray Profiling of the Increased Expression of Endo-miRNA Target Genes Resulting from the Introduction of Exo-miRNAs

**Figure 2:**
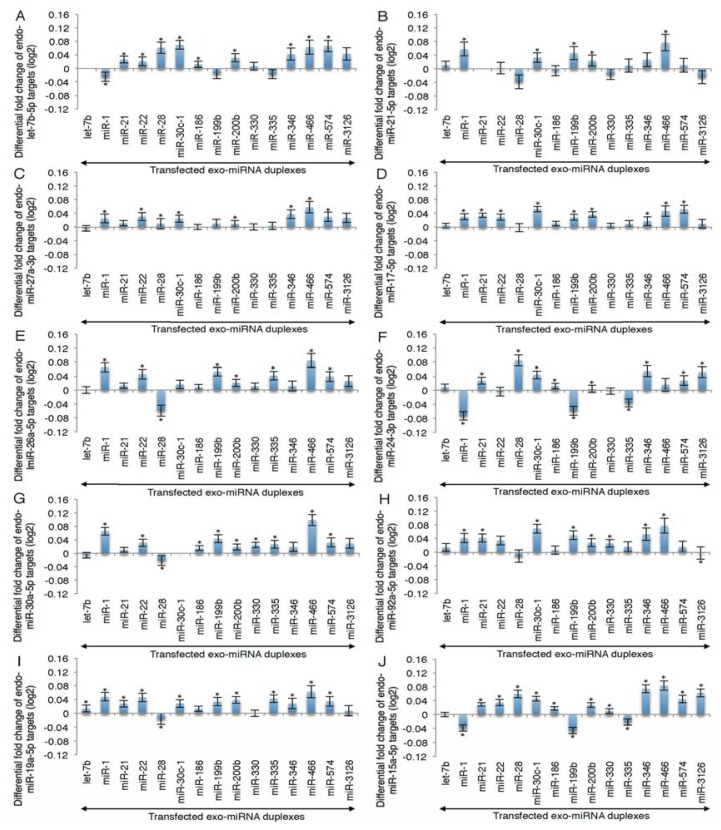

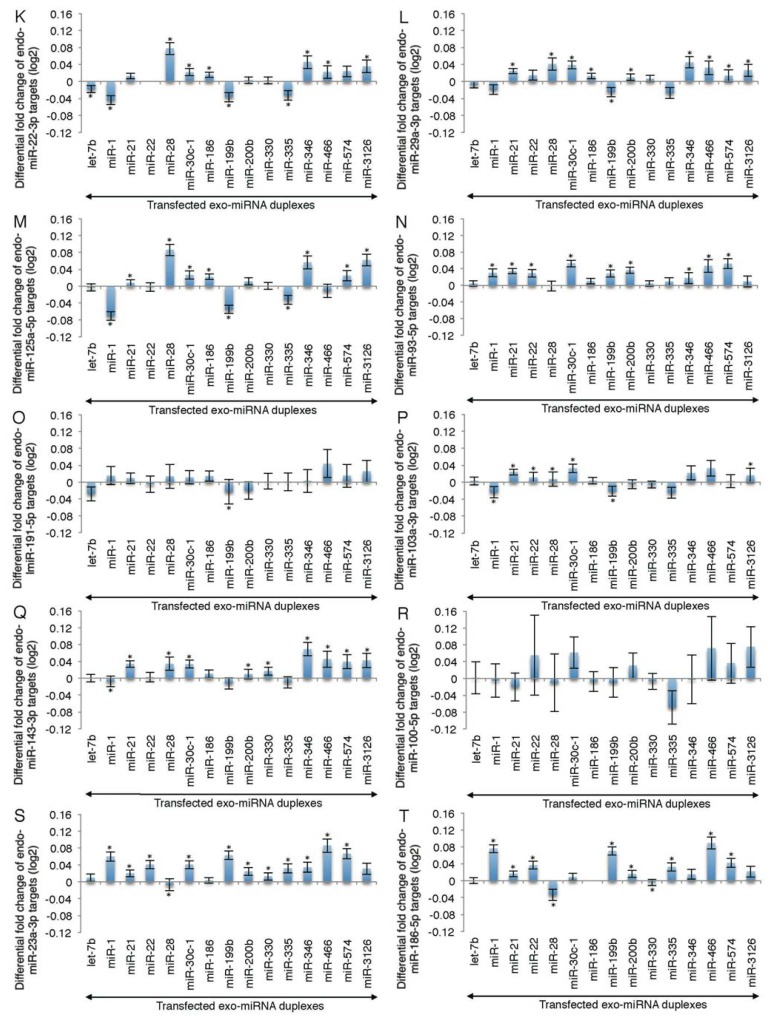

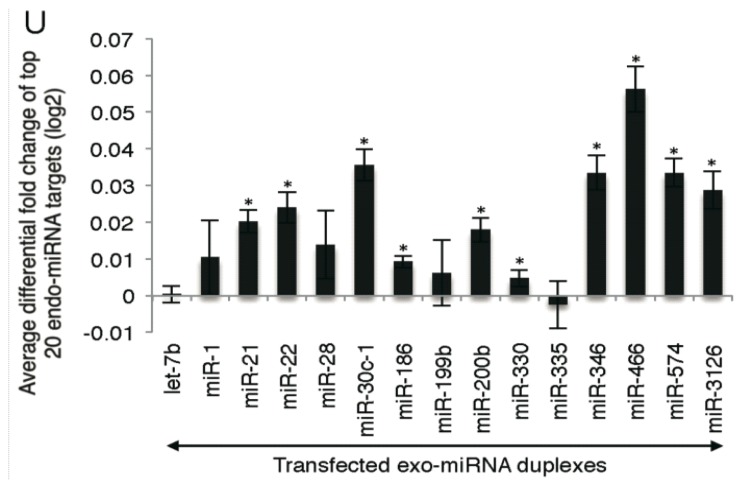
Mean fold changes of the expression levels of endo-miRNA target transcripts by the transfection of different exo-miRNAs. HeLa cells were transfected with each of fifteen exo-miRNA duplexes, log_2_ mean differential fold changes of the expression levels of seed-matched target genes of each of the top 20 endo-miRNAs were calculated. Data were shown with respect to endo-miRNAs, let-7b-5p (**A**); miR-21 (**B**); mR-27a-3p (**C**); miR-17-5p (**D**); miR-26a-5p (**E**); miR-24-3p (**F**); miR-30a-5p (**G**); miR-92a-5p (**H**); miR-19a-5p (**I**); miR-15a-5p (**J**); miR-22-3p (**K**); miR-29a-3p (**L**); miR-125a-5p (**M**); miR-93-5p (**N**); miR-191-5p (**O**); miR-103a-3p (**P**); miR-143-3p (**Q**); miR-100-5p (**R**); miR-23a-3p (**S**); and mR-186-5p (**T**); and their averaged values were shown in (**U**). Note that each exo-miRNA increased the endo-miRNA target genes in different degrees. The individual data are shown in Figures S2–S21. Data represent the mean ± SE (*p < 0.05).

**Figure 3:**
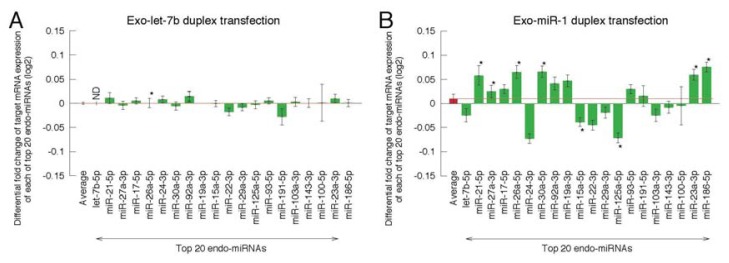

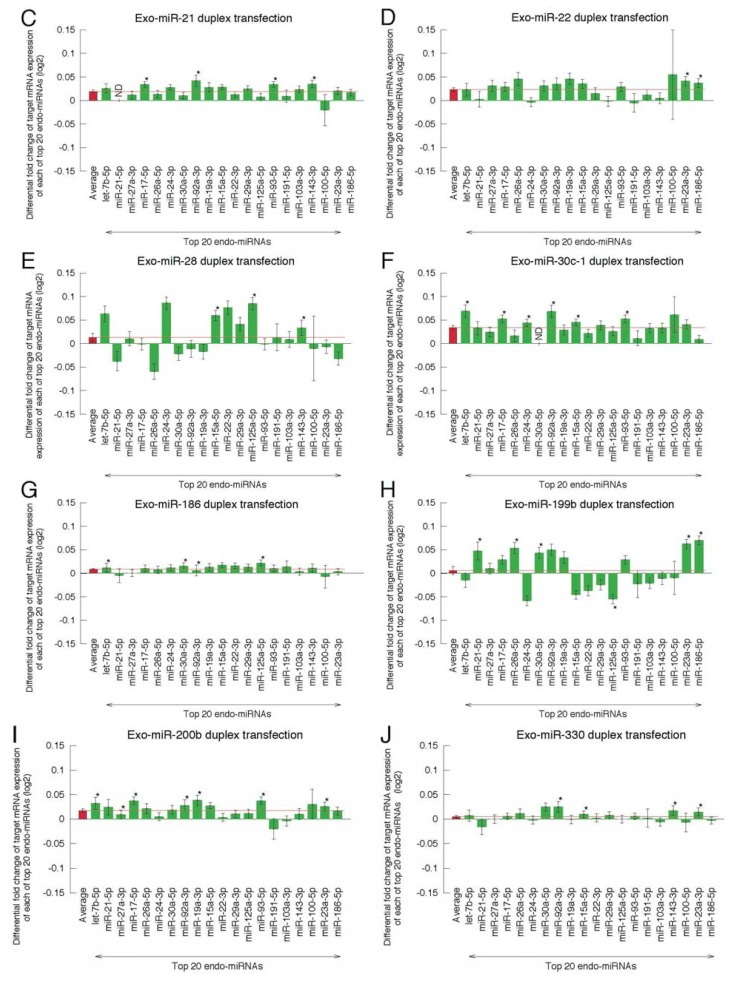

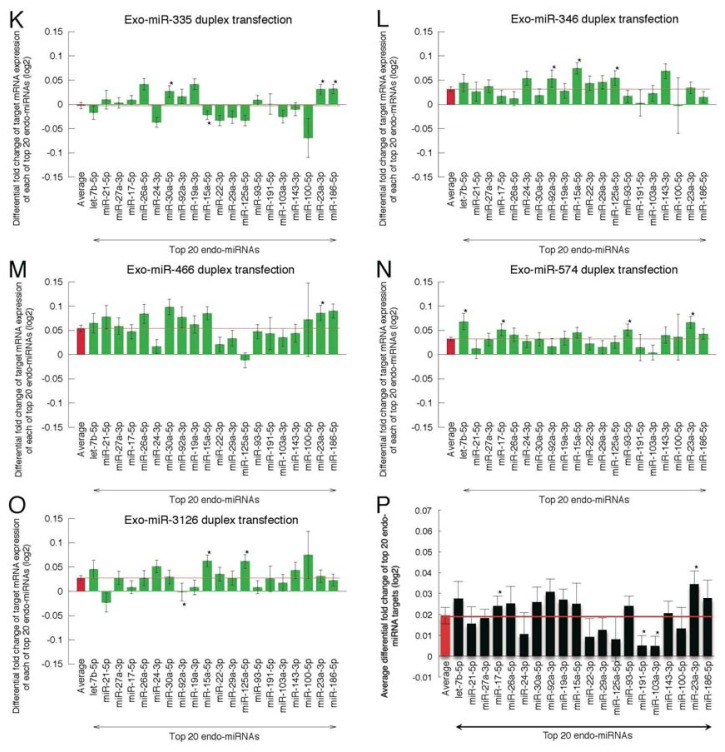
Mean fold changes of the expression levels of endo-miRNA target transcripts following the transfection of exo-miRNAs. HeLa cells were transfected with each of fifteen exo-miRNA duplexes, log_2_ mean differential fold changes of the expression levels of seed-matched target genes of each of the top 20 endo-miRNAs were calculated. Data were shown with respect to transfected exo-miRNAs, miR-7b (**A**); miR-1 (**B**); miR-21 (**C**); miR-22 (**D**); miR-28 (**E**); miR-30c-1 (**F**); miR-186 (**G**); miR-199b (**H**); miR-200b (**I**); miR-330 (**J**); miR-335 (**K**); miR-346 (**L**); miR-466 (**M**); miR-574 (**N**); miR-3126 (**O**); and their averaged values were shown in (**P**). Target genes of different endo-miRNAs are increased by exo-miRNAs in approximately comparable levels. The individual data are shown in Figures S2–S21. Data represent the mean ± SE (*p < 0.05).

Having found reduced expression of exo-miRNA target genes, we next analyzed the expression of endo-miRNA target genes. As let-7b, miR-21, miR-27a, miR-17, miR-26a, miR-24, miR-30a, miR-92a, miR-19a, miR-15a, miR-22, miR-29a, miR-125a, miR-93, miR-191, miR-103a, miR-143, miR-100, miR-23a and miR-186 are reported to be the top 20 most highly expressed miRNA families in HeLa cells [30], changes in the target gene expression of these representative miRNAs were calculated (Figures 2 and 3). In this analysis, “endo-miRNA target genes” were defined as genes with sequences complementary to endo-miRNA seed sequences (positions 2–8) in their 3′ UTRs, but not to both strands of exo-miRNA seed sequences. The “endo-miRNA non-target genes” were genes with no sequences complementary to endo-miRNA or both strands of exo-miRNA seed sequences. The MA plots and cumulative distributions were shown in Figures S2–S21. The difference values were calculated by subtracting the average fold changes (log_2_) for endo-miRNA target genes (blue line in Figures S2–S21 A~O-2) from the average value for endo-miRNA non-target genes (black line in Figures S2–S21 A-2~O-2). The expression of most of the endo-miRNA target genes was increased by the transfection of exo-miRNAs (Figures 2 and 3, Figures S2–S21).

Next, we compared changes in the expression of endo-miRNA target genes according to the types of exo-miRNAs (Figure 2). Expression changes were determined by subtracting the average log_2_ fold change for endo-miRNA target genes containing more than one target site(s) from the average value for endo-miRNA non-target genes and shown as a differential fold change (log_2_). The increases in target gene expression of endogenous top 20 miRNAs varied, but showed similar tendencies according to the kind of exo-miRNA transfected (Figure 2). Of the fifteen transfected exo-miRNAs, the exogenously transfected miR-466 duplex increased the expression levels of target genes of at least 16 endo-miRNAs (let-7b-5p, miR-21-5p, miR-27a-3p, miR-17-5p, miR-26a-5p, miR-24-3p, miR-30a-5p, miR-92a-3p, miR-19a-3p, miR-15a-5p, miR-22a-3p, miR-29a-3p, miR-93a-5p, miR-143-3p, miR-23a-3p and miR-186-5p) significantly (Figure 2A–T), and their averaged expression level was the highest (Figure 2U). The exo-miRNA duplexes, miR-21, miR-22, miR-28, miR-30c-1, miR-200b, miR-346, miR574 and miR-3126, produced significant, but modest levels of increases in the endo-miRNA target genes (Figure 2U), while exo-miRNA duplexes, let-7b, miR-1, miR-28, miR-199b and miR-335, showed low values on average (Figure 2U).

Considering another dimension of the results, changes in the target gene expression of top 20 endo-miRNAs were examined. The results clearly show that the increased ratios of target genes of any endo-miRNAs were roughly equivalent when a particular exo-miRNA was transfected (Figure 3). The average expression of seed-matched target genes of these 20 endo-miRNAs for each of 15 transfected exo-miRNA are shown in Figure 3. These results indicated that the increased fold changes of expression levels of endo-miRNA target genes were essentially determined by the kind of exo-miRNA and not by the type of endo-miRNA, although the increase of target genes of a few types of endo-miRNAs, such as miR-1 (Figure 3B), miR-28 (Figure 3E), miR-199b (Figure 3H) and miR-335 (Figure 3K), by the transfection of a given miRNA were not equivalent, and averaged values after transfection of these exo-miRNAs became low due to combining the up- and down-regulated targets (Figure 2U). The similar results, indicating the equivalent expression levels of endo-miRNA targets by most of exo-miRNAs, except for miR-1, miR-28, miR-199b and miR-335, were also observed when the increased levels were calculated for top 383 endo-miRNAs (Figure S22).

Our results were obtained from our own microarray experiments using one human cell line; it should reduce variables compared to the previous studies. To confirm the reliability of the microarray data, we analyzed 28 transcripts by quantitative RT-PCR (Figure S23). The expression levels estimated by quantitative RT-PCR were essentially identical to those obtained in the microarray analysis, with an estimated correlation coefficient of 0.83.

### 2.3. Reporter Analysis of Exo-miRNA and Endo-miRNA Target Expression in Cells Transfected with Exo-miRNA

**Figure 4:**
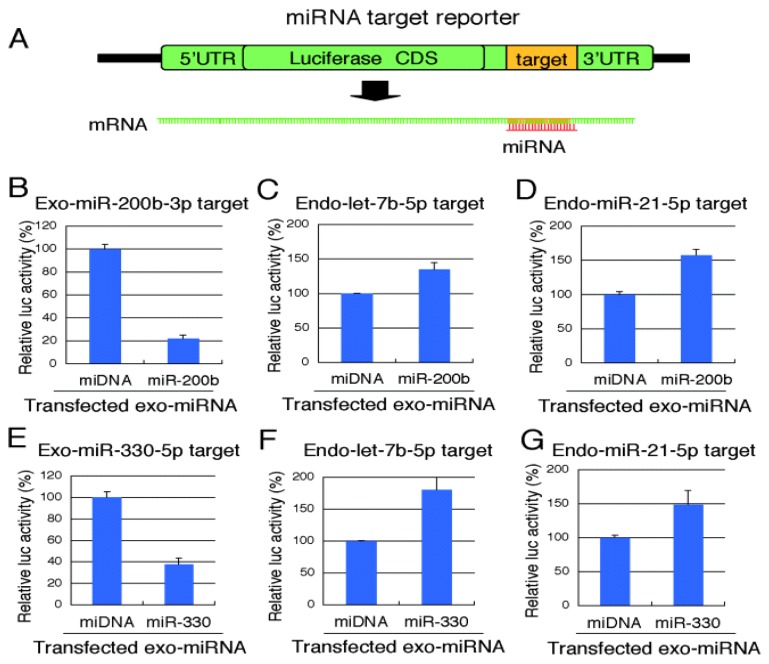
Luciferase reporter analyses of changes in the expression of exo-miRNA and endo-miRNA targets. (**A**) Schematic structure of a luciferase reporter containing a miRNA completely matched target sequence in the 3′ UTR and mRNA transcribed from the reporter. The exo-miRNAs, miR-200b (**B**–**D**) and miR-330 (**E**–**G**), respectively, were transfected into HeLa cells at 50 nM. At 24 h post-transfection, luciferase activities were measured. The luciferase activities derived from the constructs carrying exo-miRNA target sequences were decreased by the transfection of respective exo-miRNA (**B**,**E**). In contrast, the luciferase activities from the constructs carrying endo-let-7b-5p (**C**), endo-miR-21-5p (**D**,**G**) and endo-miR-7b-5p (**F**) target sequences were increased, indicating that exo-miRNAs repressed endo-miRNA silencing activities.

A part of the target genes with 3′ UTRs complementary to endo-miRNA seed sequences is not necessarily downregulated by endo-miRNAs in normal conditions. The accessibilities of endo-miRNAs to such genes are speculated to be interfered by some causes, such as binding of specific RNA binding proteins or RNA secondary structures. Thus, the changes in the expression levels of seed-complementary target genes of endo-miRNAs shown in Figures 2 and 3 might be modest or low (generally +/− 2 to 10%). To confirm the downregulation of exo-miRNA target gene expression and upregulation of endo-miRNA target gene expression resulting from transfection of exo-miRNA, we carried out reporter assays using luciferase expression constructs carrying sequences perfectly complementary to exo-miRNAs or endo-miRNAs in the 3′ UTR of the *Renilla* luciferase gene in psiCHECK-1 (Figure 4A). Even when the changes in the expression levels of seed-complementary endogenous target genes of exo-miRNAs or endo-miRNAs are small, the expression levels of luciferase reporter with perfect complementary sequences of miRNAs are expected to show remarkable effects, although the effect is not biologically relevant. Exo-miR-200b or exo-miR-330 was transfected into HeLa cells with each reporter construct and a firefly luciferase expression construct (pGL3-Cont) (internal control). A double-stranded DNA (miDNA), which mimics the miRNA structure, was used as a miRNA control. At 24 h post-transfection, the relative luciferase activity was calculated (Figure 4). Both exo-miRNAs silenced their own targets (Figure 4B,E). In contrast, the luciferase activities of psiCHECK-1 constructs containing the endo-miRNA target sequences apparently increased (Figure 4C,D,F,G). These results suggest that exo-miRNAs interfered with endo-miRNA silencing activity by competing for the RNA silencing machinery downstream of Exportin-5 probably replacing with endo-miRNAs in the RISC, thereby leading to increased expression of endo-miRNA target genes.

### 2.4. Exo-miRNA Targets with Short 3′ UTRs Are Efficiently Downregulated and Endo-miRNA Targets with Long 3′UTRs Are Efficiently Upregulated by the Introduction of Exo-miRNA Duplex

**Figure 5:**
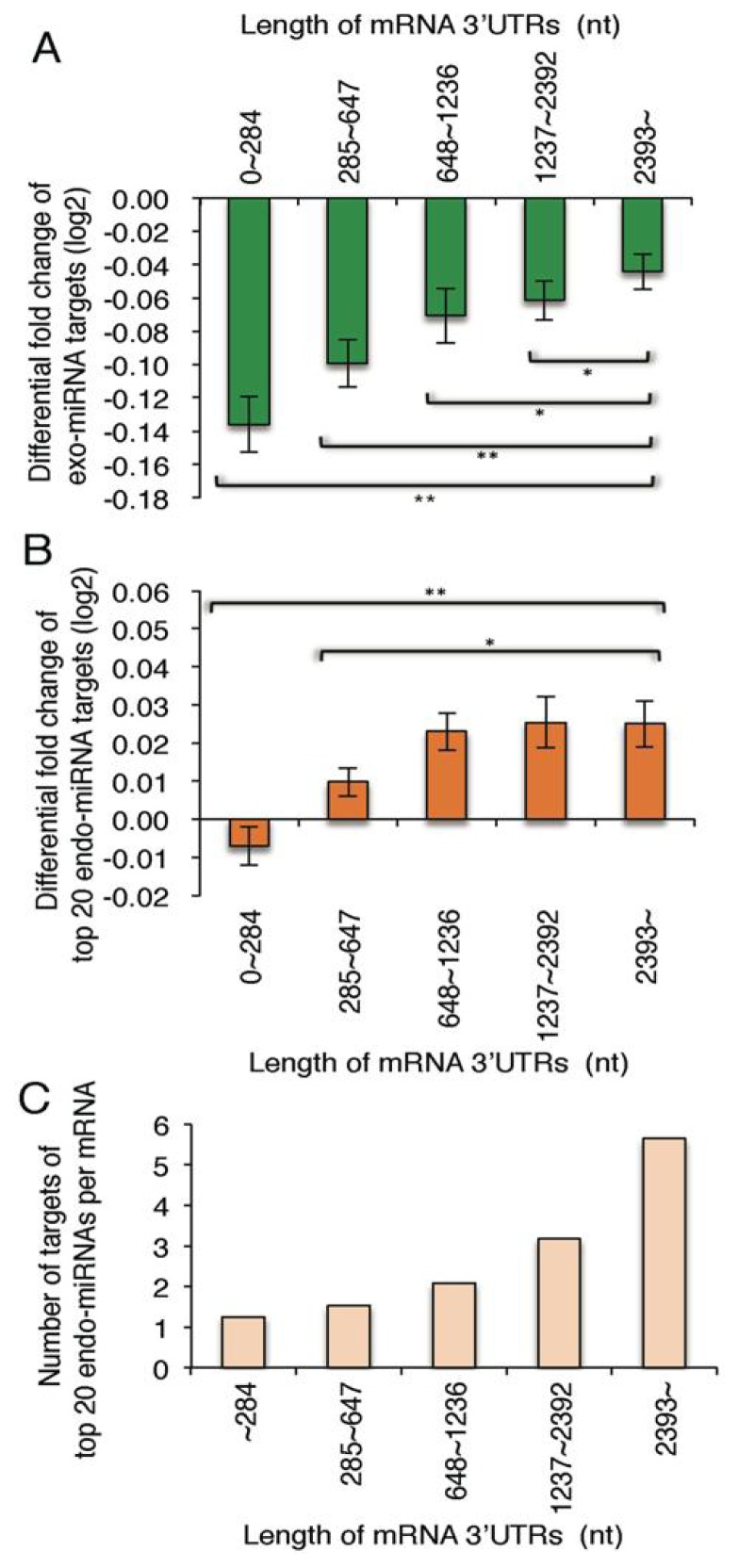
Mean fold changes of target genes of exo-miRNAs and endo-miRNAs according to the 3′ UTR lengths. Differential fold changes (log_2_) of expression levels of exo-miRNA targets (**A**); and endo-miRNA targets (**B**); and the number of top 20 endo-miRNA target sites (**C**) according to the lengths of 3′ UTRs. The averaged numbers of exo-miRNA target sites are 53, 137, 210, 250 and 149 (**A**), and those of endo-miRNA target sites are 53, 134, 185, 184 and 84 (**B**) in the 3′ UTRs of 0–284, 285–647, 648–1236, 1237–2392 and 2393~nucleotides (nts). Each fraction contains the same number of mRNAs registered in the RefSeq database.

Among exo-miRNA target genes, the genes with short 3′ UTRs were more efficiently downregulated than those with long 3′ UTRs by the transfection of exo-miRNAs (Figure 5A), consistent with the previous report [31]. In this analysis, we used the fold change values of exo-miRNA targets with 3′ UTRs complementary to seed sequence of either one strand of exo-miRNA duplex: let-7b-5p, miR-1, miR-21-5p, miR-22-5p, miR-28-5p, miR-30c-5p, miR-186-5p, miR-199b-5p, miR-200b-3p, miR-330-5p, miR-335-5p, miR-346, miR-466, miR-574-5p and miR-3126-5p. In contrast, the top 20 endo-miRNA targets, which have no exo-miRNA target sites, were found to be more strongly upregulated, according to the increase of the 3′ UTR lengths (Figure 5B). It was presumed that a large number of endo-miRNA target sites are situated in the long 3′ UTRs, but small in the short 3′ UTRs; then, the genes with long 3′ UTRs might be strongly repressed by endo-miRNAs in the normal condition, but those with short 3′ UTRs might be repressed weakly. So, we calculated the number of top 20 endo-miRNA target sites in the various length of mRNA 3′ UTRs. As expected, a large number of target sites of top 20 endo-miRNAs were found in the long 3′ UTRs, but a small number of them were in the short 3′ UTRs (Figure 5C), indicating that the genes with long 3′ UTRs should be strongly repressed by endo-miRNAs compared to those with short 3′ UTRs in the normal condition. However, once exo-miRNAs are transfected into the cells, a part of the endo-miRNAs loaded on the RISCs should be replaced with exo-miRNAs. As a result, the constant downregulation of endo-miRNA targets by endo-miRNAs might be cancelled, and the expression of these genes are upregulated. Thus, the changes of expression levels of the exo-miRNA target genes might be elaborately regulated by the endo-miRNAs pre-situated in the RISCs.

### 2.5. Highly Expressed Exo-miRNA Targets Are Efficiently Downregulated and Endo-miRNA Targets with Low Expression Levels Are Efficiently Upregulated by the Transfection of the Exo-miRNA Duplex

**Figure 6:**
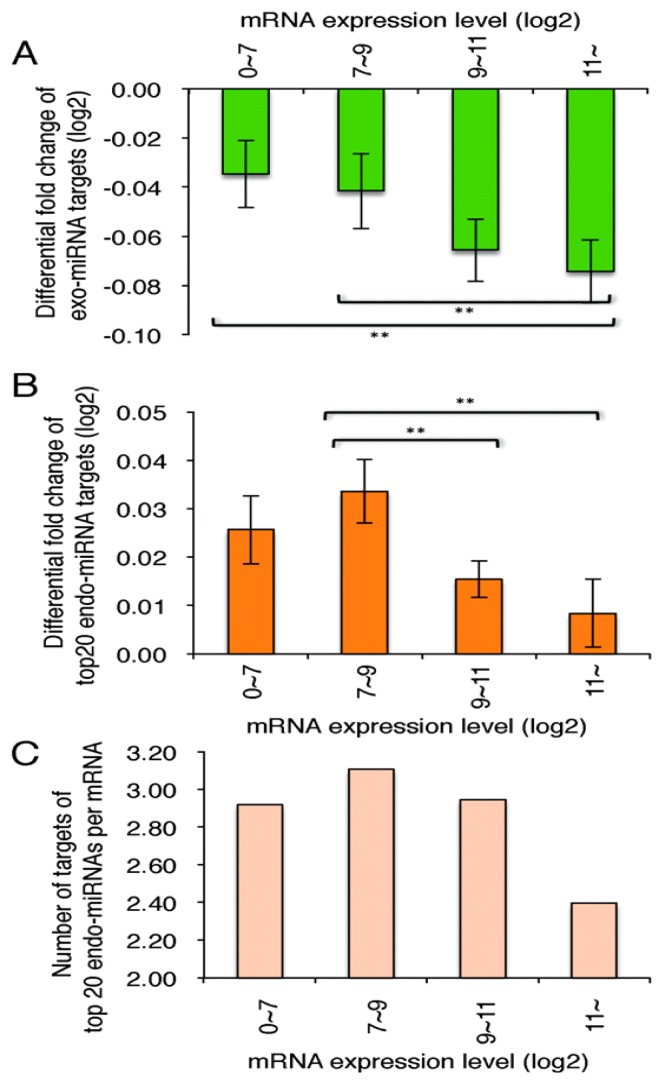
Mean fold changes of target genes of exo-miRNAs and endo-miRNAs, according to the expression levels. Differential fold changes (log_2_) of target gene expression levels of exo-miRNAs (**A**) and endo-miRNAs (**B**) and the number of the top 20 endo-miRNA target sites (**C**), according to the expression levels of exo-miRNA targets. The averaged numbers of exo-miRNA target sites are 130, 217, 237 and 215 (**A**), and those of endo-miRNA target sites are 92, 164, 176 and 202 (**B**) in the 3′ UTRs of genes with differential fold changes of 0~7, 7~9, 9~11 and 11~.

Among exo-miRNA targets, the genes with high expression levels in the normal conditions were more efficiently downregulated by the transfection of exo-miRNAs compared to those with low expression levels (Figure 6A). The results showed good agreement with the previous study [31]. In contrast, the endo-miRNA targets with low expression levels in the normal conditions were uncovered to be intensively upregulated by the exo-miRNA transfection, and those with high expression levels were not upregulated efficiently (Figure 6B). So, we investigated the number of endo-miRNA target sites in gene with different expression levels. As a result, a large number of target sites were found in the mRNAs with low expression levels, but a small number of the sites were detected in the mRNAs with high expression levels (Figure 6C), suggesting that many genes with low expression levels might be downregulated by endo-miRNAs, but those with high expression levels are not repressed by endo-miRNAs in the normal condition. Thus, the changes of expression levels of the exo-miRNA target genes might be also suitably regulated by the endo-miRNAs pre-situated in the RISCs.

## 3. Experimental Section

### 3.1. Cell Culture and miRNA Synthesis

Human HeLa cells were cultured and used in reporter assays and microarray analyses. Cells were cultured at 37 °C in Dulbecco’s modified Eagle’s Medium (Invitrogen, Carlsbad, NM, USA), supplemented with 10% heat-inactivated fetal bovine serum (Sigma, St. Louis, MO, USA). They were plated on 24-well culture plates (1 × 10^5^ cells/mL/well) 24 h prior to transfection. Transfection was carried out using Lipofectamine 2000 (Invitrogen, Carlsbad, NM, USA). Each RNA strand of the miRNA duplex was chemically synthesized (Sigma, St. Louis, MO, USA) and annealed to form the duplex structures the same as those shown in miRBase [28]. The sequences of the synthetic miRNAs (let-7b, miR-1, miR-21, miR-22, miR-28, miR-30c-1, miR-186, miR-199b, miR-200b, miR-330, miR-335, miR-346, miR-466, miR-574 and miR-3126) are listed in Table S1.

### 3.2. Construction of Luciferase Reporters

All of the reporter plasmids constructed were derivatives of psiCHECK-1 (Promega, Fitchburg, WI, USA). Oligonucleotides with target sequences completely matched to each miRNA strand (cm-target) were chemically synthesized with cohesive *Xho*I/*Eco*RI ends (Table S2). They were then inserted into the corresponding restriction sites of psiCHECK-1 to generate miRNA cm-targets (miR-200b-3p target, miR-7b-5p target, miR-21-5p target and miR-330-5p target). Each of the inserted targets was expressed as part of the 3′ UTR region of *Renilla* luciferase mRNA in transfected cells.

HeLa cells growing in 24-well plates were transfected simultaneously with miRNA target (100 ng), pGL3-Control (Promega, 0.5 μg) and miRNA (50 nM). The cells were harvested 24 h post-transfection and the relative luciferase activity (*Renilla* luc activity/firefly luc activity) was determined using a Dual-Luciferase Reporter Assay System (Promega, Fitchburg, WI, USA). The pGL3-Control encoding firefly luciferase served as a control for the calculation of relative luciferase activity for miRNAs.

### 3.3. Microarray Analysis

HeLa cells (1 × 10^5^ cells/mL) were transfected with 50 nM of each of 15 miRNA duplexes. At 24 h post-transfection, total RNA was purified using an RNeasy Kit (Qiagen, Hilden, Germany). The steps were repeated four times and RNA quality assessed using a NanoDrop 2000 spectrophotometer (Thermo Scientific, Waltham, MA, USA) and a Bioanalyzer (Agilent, Santa Clara, CA, USA). RNAs recovered independently were mixed equally for cDNA synthesis using an Agilent One Color Spike Mix Kit (Agilent, Santa Clara, CA, USA). Cy3-labeled cRNA was synthesized using a Quick Amp Labeling Kit (Agilent, Santa Clara, CA, USA) and was hybridized to an Agilent Whole Human Genome Microarray (4 × 44 K multi-pack format), according to the manufacturer’s protocol. RNA from mock-transfected cells treated with transfection reagent in the absence of miRNA was used as a control. Transcript expression values were calculated using Microarray Suite 5.0 (MAS5: Affymetrix, Santa Clara, CA, USA) [32] with quantile normalization [33]. To identify transcripts whose expression was upregulated or downregulated, the cumulative distribution of expression changes for transcripts containing the site was compared with that for transcripts with no canonical site. NCBI’s Reference Sequence (RefSeq) was used to identify mRNAs with sequences complementary to the seed regions of the transfected miRNAs. Data are presented as an MA plot (M = intensity ratio, A = average intensity) and a cumulative frequency distribution. Changes in expression are shown as fold changes (log_2_).

### 3.4. Quantitative RT-PCR

Total RNA was reverse-transcribed using a Transcriptor High Fidelity cDNA Synthesis kit (Roche, Basel, Switzerland). The resultant cDNA samples were incubated with FastStart Universal SYBR Green Master (Roche, Basel, Switzerland) at 95 °C for 10 min, followed by PCR amplification. PCR product levels were monitored using an ABI PRISM 7000 sequence detection system and analyzed with ABI PRISM 7000 SDS software (Applied Biosystems). The expression of each target gene was first normalized to that of β-actin and then to the mock-transfection control. The primer sets used are listed in Table S3.

## 4. Conclusions

In this study, we quantified the changes in expression levels of endo-miRNA target genes resulting from the transfection of exo-miRNA duplexes. The expression levels of endo-miRNA target genes with seed-complementary sequences were increased by the transfection of exo-miRNA duplexes, while exo-miRNA target gene expression was reduced (Figures 1 – 3 and Figures S1–S3). These results suggest that exo-miRNA duplex transfected into the cells may compete with endo-miRNAs for the RISC, which may be saturated with endo-miRNAs under normal conditions in HeLa cells.

**Figure 7:**
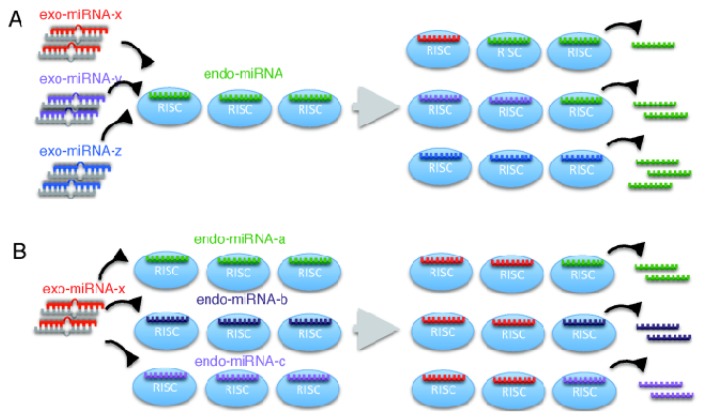
Predicted model for the RNA-induced silencing complexes (RISCs) replacement. Different types of exo-miRNAs transfected into cells may be replaced with single-stranded endo-miRNAs loaded on the RISC with different efficiencies (**A**); however, different types of endo-miRNAs may be replaced with a given exo-miRNAs with similar efficiencies (**B**).

In miRNA-mediated gene silencing, the structure of the RNA-protein complex is known to be altered [23]. The miRNA duplex in RISC loading complex is unwound, yielding single-stranded RNA, which is loaded onto the RISC and recognizes target mRNAs through base-pairing in the seed region [4–6]. Our results indicate that the expression of the target genes of a given endo-miRNA differed according to the exo-miRNA duplex that was transfected (Figures 2 and 7A), whereas with a given exo-miRNA duplex, the fold changes in the target gene expression of the endo-miRNAs examined were mostly equivalent, except for a limited types of exo-miRNAs (miR-1, miR-28, miR-199b and miR-335), despite differences in the structures and sequences of the endo-miRNA duplexes (Figure 3 and Figure 7B). One of the possible explanations of these results is that the RISC exchange reaction might be occurred associated with single-stranded endo-miRNAs and not endo-miRNA duplexes or target-paired endo-miRNAs (Figure 7), because double-stranded endo-miRNAs in the RISC might not be replaced with exo-miRNAs at similar levels, due to their different structures and sequences. Most miRNA-RISCs might be in the form of single-stranded miRNAs in RISCs, so as to be readily replaced by double-stranded miRNAs.

Furthermore, it was apparently revealed that endo-miRNAs constantly repress the expression of endogenous mRNAs with endo-miRNA target sites, and their repression is probably relieved by the replacement of endo-miRNAs on the RISC by the exo-miRNAs transfected (Figures 5 and 6). Thus, global gene expression by endogenous miRNAs might be fluctuated by the transfection of exo-miRNAs or the increase of expression levels of endo-miRNAs. Competition similar to that shown here between exo-miRNA duplexes and endo-miRNAs may also occur among newly transcribed endo-miRNAs and may provide a mechanism for orchestrating cellular programs.

## Supplementary Information



## Supplementary Information
