# Supplementary material for: Fluctuation of Global Gene Expression by Endogenous miRNA Response to the Introduction of an Exogenous miRNA
Source: Int J Mol Sci. 2013 May 27;14(6):11171–89. doi: 10.3390/ijms140611171 (PMC3709726; doi:10.3390/ijms140611171)

Supplementary Information

Figure S1. Microarray analysis of the expression of target genes of the transfected exo-miRNAs
HeLa cells transfected with each of the fifteen exo-miRNA duplexes were subjected to microarray profiling. Data are presented as MA plots (A1-O1, A3-O3) and cumulative distributions (A2-O2, A4-O4) of seed-matched target genes of let-7b-5p (A-1 and A-2) and let-7b-3p (A-3 and A-4), miR-1 (B-1 and B-2), miR-21-5p (C-1 and C-2) and miR-21-3p (C-3 and C-4), miR-22-5p (D-1 and D-2) and miR-22-3p (D-3 and D-4), miR-28-5p (E-1 and E-2) and miR-28-3p (E-3 and E-4), miR-30c-5p (F-1 and F-2) and miR-30c-3p (F-3 and F-4), miR-186-5p (G-1 and G-2) and miR-186-3p (G-3 and G-4), miR-199b-5p (H-1 and H-2) and miR-199b-3p (H-3 and H-4), miR-200b-5p (I-1 and I-2) and miR-200b-3p (I-3 and I-4), miR-330-5p (J-1 and J-2) and miR-330-3p (J-3 and J-4), miR-335-5p (K-1 and K-2) and miR-335-3p (K-3 and K-4), miR-346 (L-1 and L-2), miR-446 (M-1 and M-2), miR-574-5p (N-1 and N-2) and miR-574-3p (N-3 and N-4), and miR-3126-5p (O-1 and O-2) and miR-3126-3p (O-3 and O-4). In MA plots, the vertical axis indicates the change in gene expression (log_2 fold change relative to mock-transfected cells), and the horizontal axis indicates the mean signal intensity for the probes for each gene before and after miRNA transfection. The numbers of target sites in the 3' UTR are indicated by dots of different colors: navy blue, ≥4 seed-matched target sites in a single 3' UTR; dark blue, 3 target sites; blue, 2 sites; light blue, 1 site; gray, no seed-matched sites. (A-2~O-2, A-4~O-4). In cumulative distributions, blue and black lines indicate the cumulative fraction of seed-matched target transcripts (≥1 seed-matched target sites) and all transcripts, respectively. ND; Not determined.

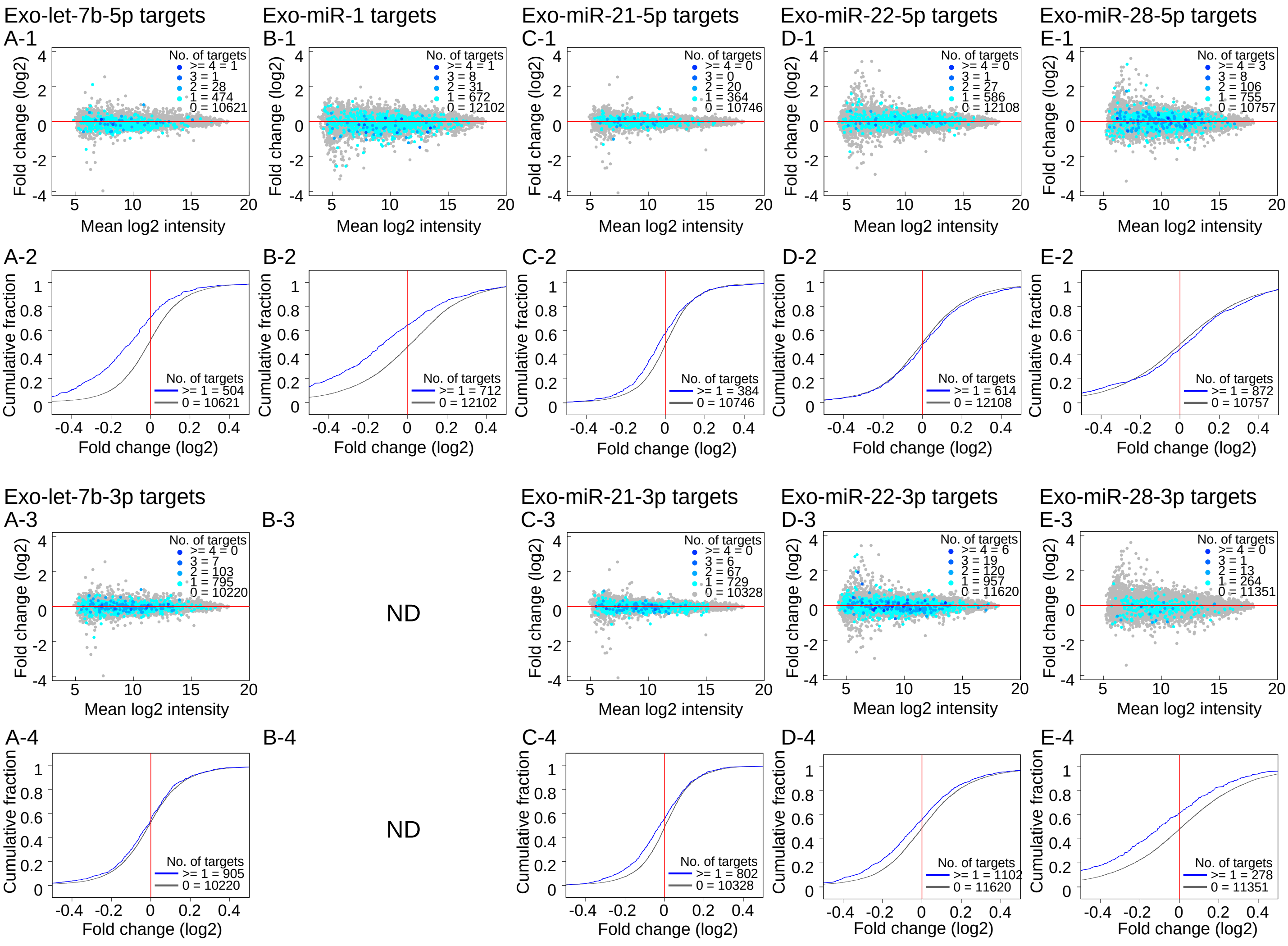

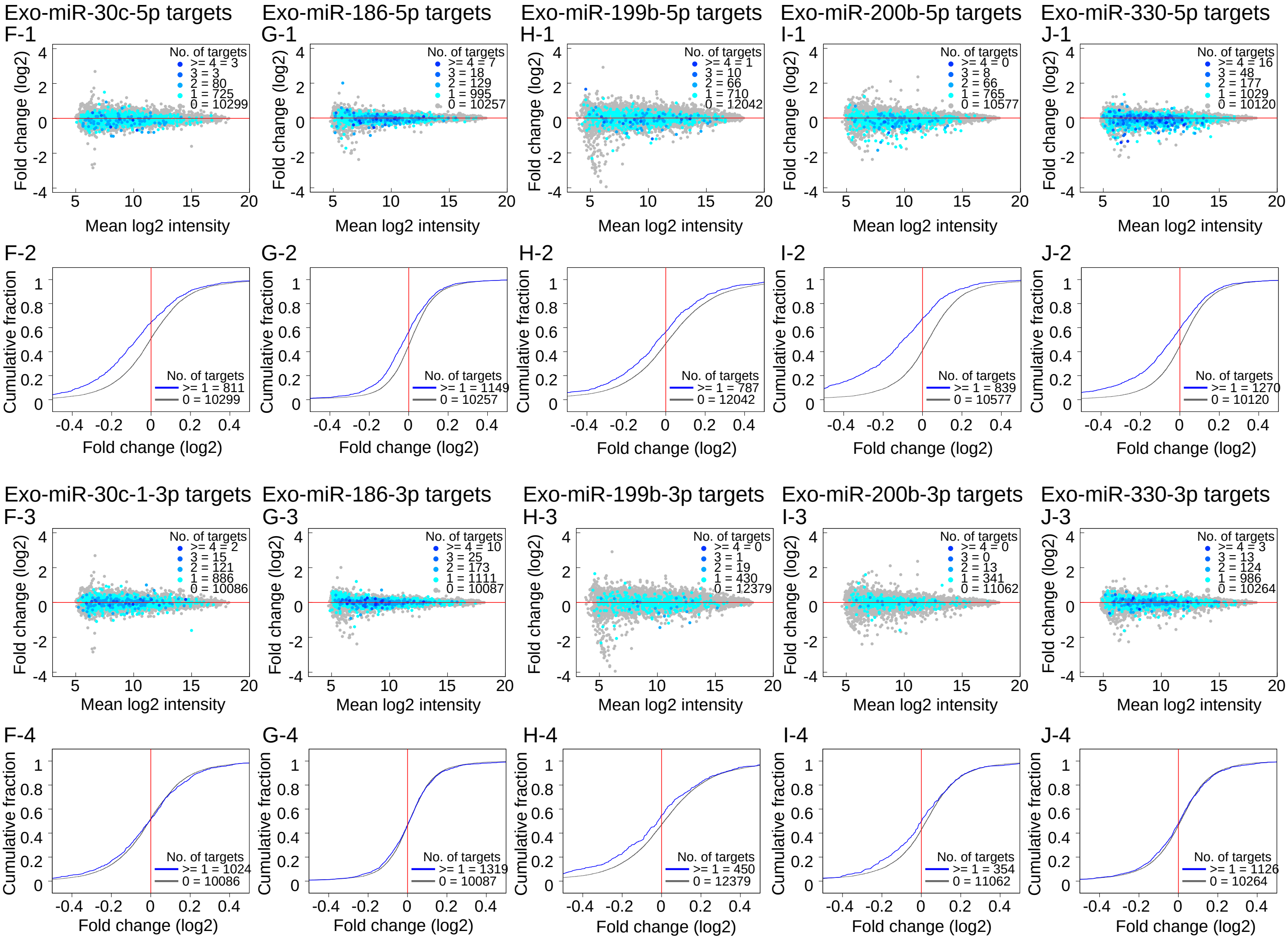

Exo-miR-30c-5p targets
F-1
Exo-miR-186-5p targets
G-1
Exo-miR-199b-5p targets
H-1
Exo-miR-200b-5p targets
I-1
Exo-miR-330-5p targets
J-1
Fold change (log2)
Mean log2 intensity
No. of targets
>= 4 = 3
3 = 3
2 = 80
1 = 725
0 = 10299
>= 4 = 7
3 = 18
2 = 129
1 = 995
0 = 10257
>= 4 = 1
3 = 10
2 = 66
1 = 710
0 = 12042
>= 4 = 0
3 = 8
2 = 66
1 = 765
0 = 10577
>= 4 = 16
3 = 48
2 = 177
1 = 1029
0 = 10120
F-2
G-2
H-2
I-2
J-2
Cumulative fraction
Fold change (log2)
>= 1 = 811
0 = 10299
>= 1 = 1149
0 = 10257
>= 1 = 787
0 = 12042
>= 1 = 839
0 = 10577
>= 1 = 1270
0 = 10120
Exo-miR-30c-1-3p targets
F-3
Exo-miR-186-3p targets
G-3
Exo-miR-199b-3p targets
H-3
Exo-miR-200b-3p targets
I-3
Exo-miR-330-3p targets
J-3
>= 4 = 2
3 = 15
2 = 121
1 = 886
0 = 10086
>= 4 = 10
3 = 25
2 = 173
1 = 1111
0 = 10087
>= 4 = 0
3 = 1
2 = 19
1 = 430
0 = 12379
>= 4 = 0
3 = 0
2 = 13
1 = 341
0 = 11062
>= 4 = 3
3 = 13
2 = 124
1 = 986
0 = 10264
F-4
G-4
H-4
I-4
J-4
>= 1 = 1024
0 = 10086
>= 1 = 1319
0 = 10087
>= 1 = 450
0 = 12379
>= 1 = 354
0 = 11062
>= 1 = 1126
0 = 10264

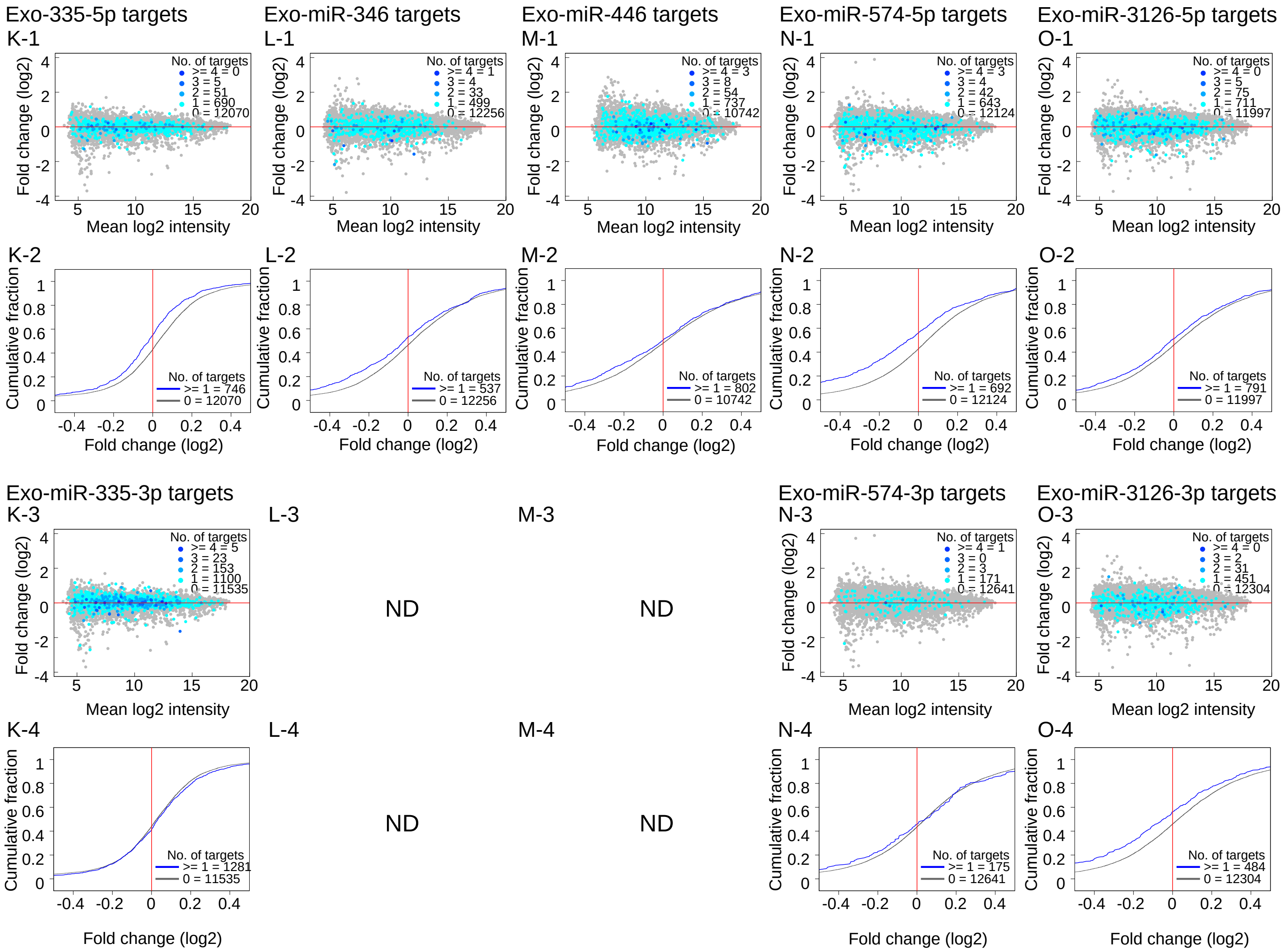

Exo-335-5p targets
K-1
No. of targets
>= 4 = 0
3 = 5
2 = 51
1 = 690
0 = 12070
Fold change (log2)
Mean log2 intensity
Exo-miR-346 targets
L-1
No. of targets
>= 4 = 1
3 = 4
2 = 33
1 = 499
0 = 12256
Exo-miR-446 targets
M-1
No. of targets
>= 4 = 3
3 = 8
2 = 54
1 = 737
0 = 10742
Exo-miR-574-5p targets
N-1
No. of targets
>= 4 = 3
3 = 4
2 = 42
1 = 643
0 = 12124
Exo-miR-3126-5p targets
O-1
No. of targets
>= 4 = 0
3 = 5
2 = 75
1 = 711
0 = 11997
K-2
Cumulative fraction
Fold change (log2)
No. of targets
>= 1 = 746
0 = 12070
L-2
No. of targets
>= 1 = 537
0 = 12256
M-2
No. of targets
>= 1 = 802
0 = 10742
N-2
No. of targets
>= 1 = 692
0 = 12124
O-2
No. of targets
>= 1 = 791
0 = 11997
Exo-miR-335-3p targets
K-3
No. of targets
>= 4 = 5
3 = 23
2 = 153
1 = 1100
0 = 11535
L-3
ND
M-3
ND
Exo-miR-574-3p targets
N-3
No. of targets
>= 4 = 1
3 = 0
2 = 3
1 = 171
0 = 12641
Exo-miR-3126-3p targets
O-3
No. of targets
>= 4 = 0
3 = 2
2 = 31
1 = 451
0 = 12304
K-4
No. of targets
>= 1 = 1281
0 = 11535
L-4
ND
M-4
ND
N-4
No. of targets
>= 1 = 175
0 = 12641
O-4
No. of targets
>= 1 = 484
0 = 12304

Figure S2. Microarray analysis of the expression of target genes of the endo-miRNA, let-7b-5p Microarray analysis of endo-let-7b-5p target gene expression in cells transfected with exo-let-7b (A), exo-miR-1 (B), exo-miR-21 (C), exo-miR-22 (D), exo-miR-28 (E), exo-miR-30c-1 (F), exo-miR-186 (G), exo-miR-199b (H), exo-miR-200b (I), exo-miR-330 (J), exo-miR-335 (K), exo-miR-346 (L), exo-miR-446 (M), exo-miR-574 (N), exo-miR-3126 (O). Data are presented as MA plots (A-1~O-1) and cumulative distributions (A-2~O-2) of seed-matched target genes of let-7b-5p.

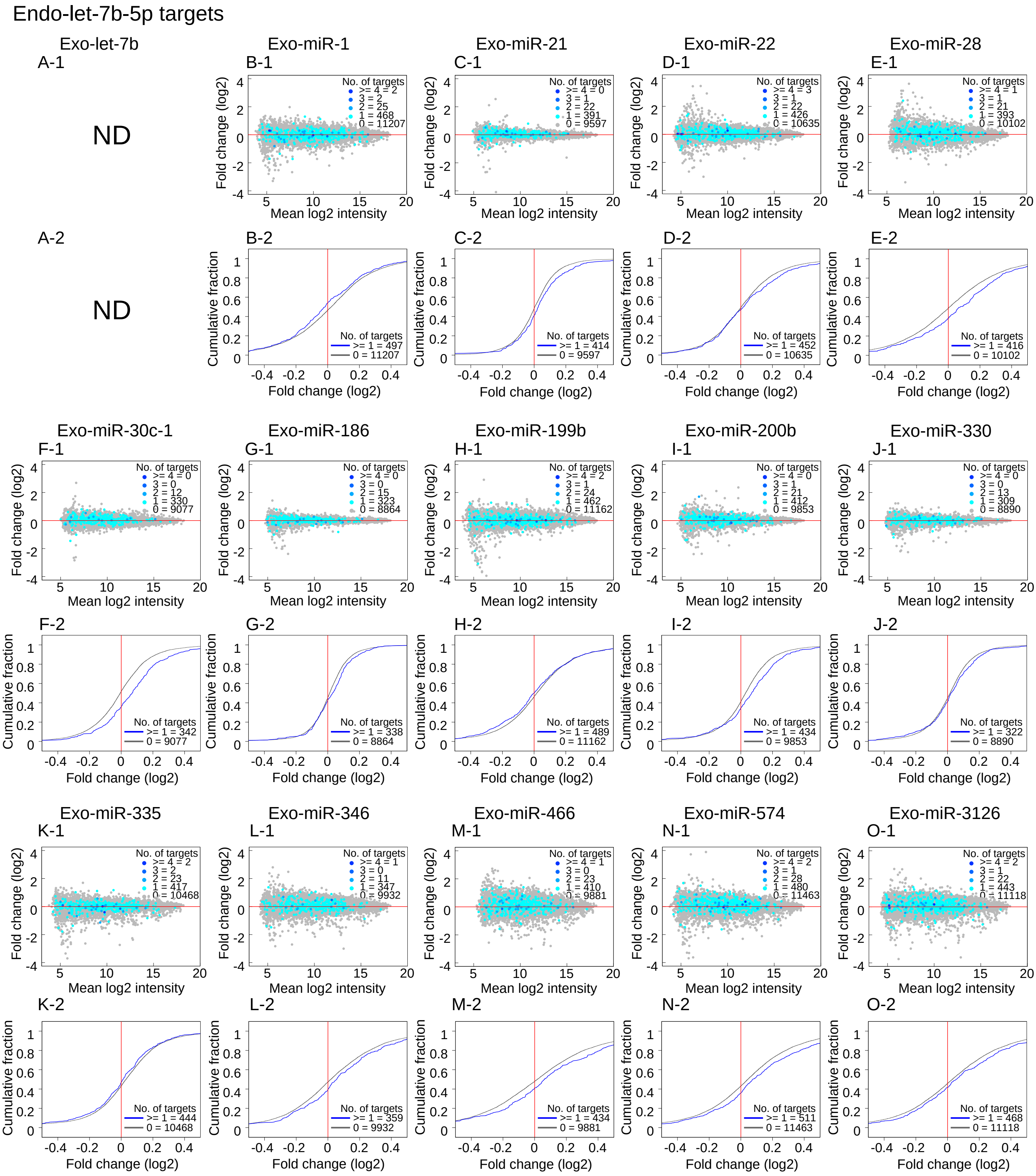

Figure S3. Microarray analysis of the expression of target genes of the endo-miRNA, miR-21-5p Microarray analysis of endo-miR-21-5p target gene expression in cells transfected with exo-let-7b (A), exo-miR-1 (B), exo-miR-21 (C), exo-miR-22 (D), exo-miR-28 (E), exo-miR-30c-1 (F), exo-miR-186 (G), exo-miR-199b (H), exo-miR-200b (I), exo-miR-330 (J), exo-miR-335 (K), exo-miR-346 (L), exo-miR-446 (M), exo-miR-574 (N), exo-miR-3126 (O). Data are presented as MA plots (A-1~O-1) and cumulative distributions (A-2~O-2) of seed-matched target genes of miR-21-5p.

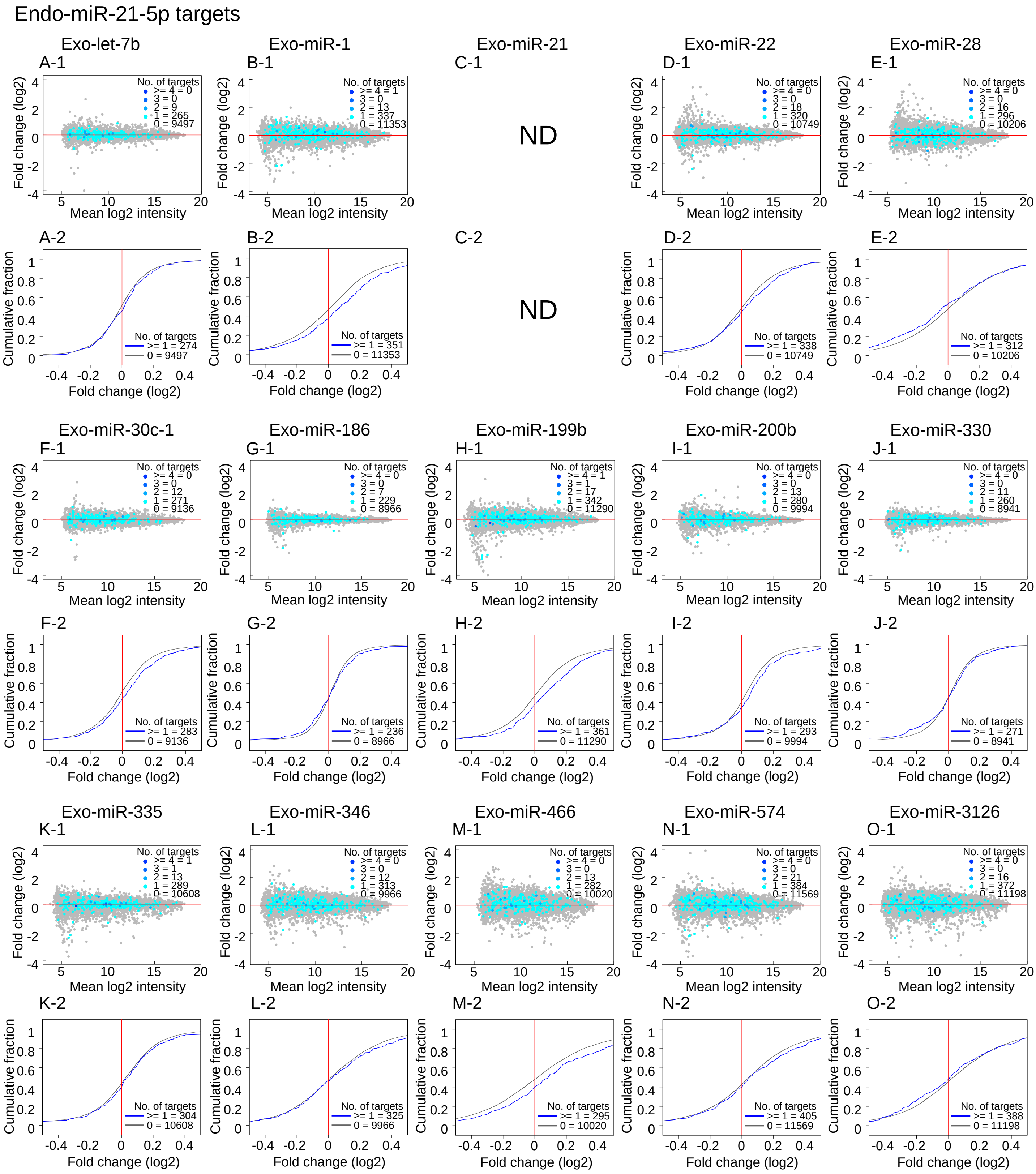

Figure S4. Microarray analysis of the expression of target genes of the endo-miRNA, miR-27a-3p Microarray analysis of endo-miR-27a-3p target gene expression in cells transfected with exo-let-7b (A), exo-miR-1 (B), exo-miR-21 (C), exo-miR-22 (D), exo-miR-28 (E), exo-miR-30c-1 (F), exo-miR-186 (G), exo-miR-199b (H), exo-miR-200b (I), exo-miR-330 (J), exo-miR-335 (K), exo-miR-346 (L), exo-miR-446 (M), exo-miR-574 (N), exo-miR-3126 (O). Data are presented as MA plots (A-1~O-1) and cumulative distributions (A-2~O-2) of seed-matched target genes of miR-27a-3p.

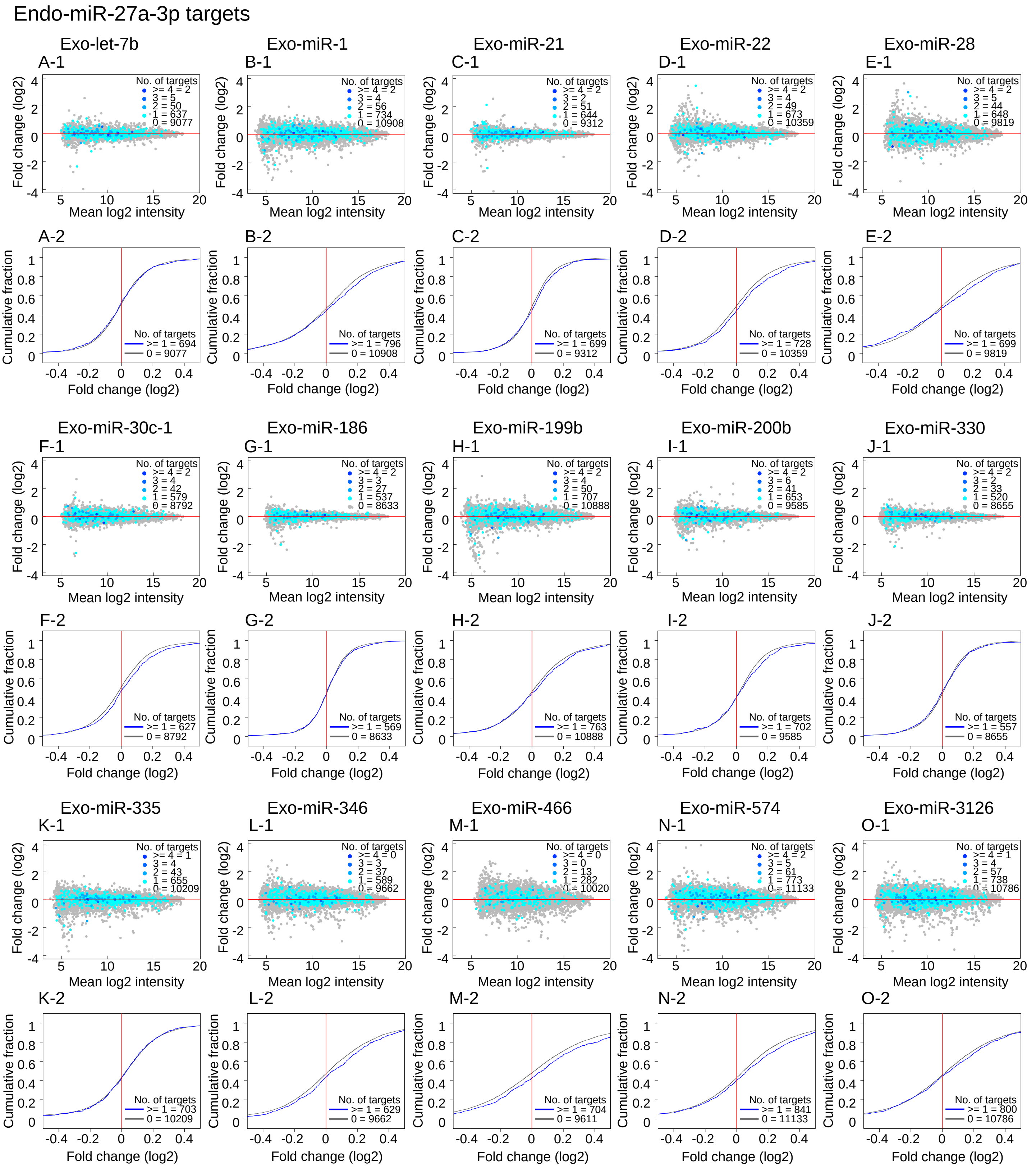

Figure S5. Microarray analysis of the expression of target genes of the endo-miRNA, miR-17-5p Microarray analysis of endo-miR-17-5p target gene expression in cells transfected with exo-let-7b (A), exo-miR-1 (B), exo-miR-21 (C), exo-miR-22 (D), exo-miR-28 (E), exo-miR-30c-1 (F), exo-miR-186 (G), exo-miR-199b (H), exo-miR-200b (I), exo-miR-330 (J), exo-miR-335 (K), exo-miR-346 (L), exo-miR-446 (M), exo-miR-574 (N), exo-miR-3126 (O). Data are presented as MA plots (A-1~O-1) and cumulative distributions (A-2~O-2) of seed-matched target genes of miR-17-5p.

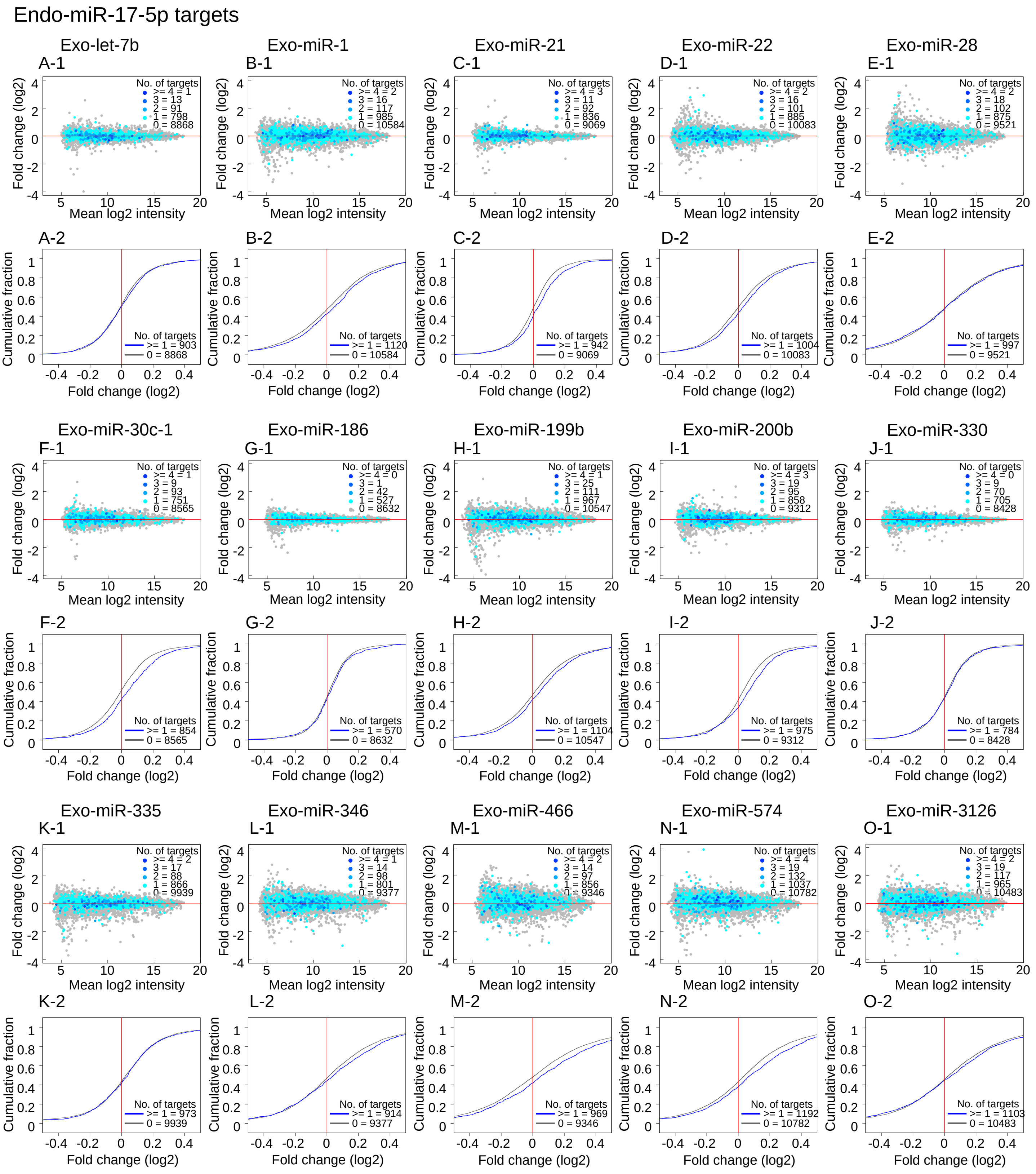

Figure S6. Microarray analysis of the expression of target genes of the endo-miRNA, miR-26a-5p Microarray analysis of endo-miR-26a-5p target gene expression in cells transfected with exo-let-7b (A), exo-miR-1 (B), exo-miR-21 (C), exo-miR-22 (D), exo-miR-28 (E), exo-miR-30c-1 (F), exo-miR-186 (G), exo-miR-199b (H), exo-miR-200b (I), exo-miR-330 (J), exo-miR-335 (K), exo-miR-346 (L), exo-miR-446 (M), exo-miR-574 (N), exo-miR-3126 (O). Data are presented as MA plots (A-1~O-1) and cumulative distributions (A-2~O-2) of seed-matched target genes of miR-26a-5p.

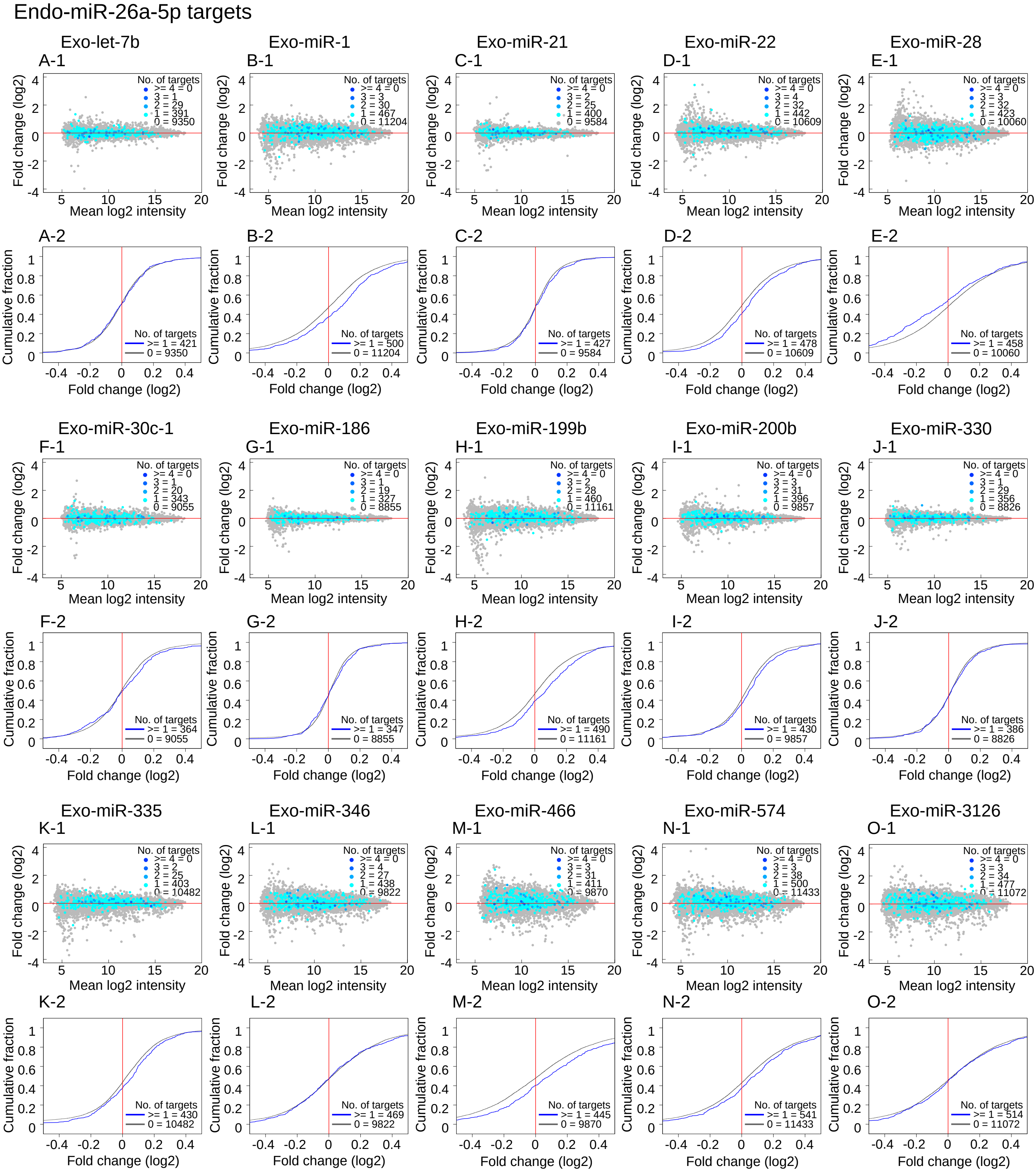

Figure S7. Microarray analysis of the expression of target genes of the endo-miRNA, miR-24-3p Microarray analysis of endo-miR-24-3p target gene expression in cells transfected with exo-let-7b (A), exo-miR-1 (B), exo-miR-21 (C), exo-miR-22 (D), exo-miR-28 (E), exo-miR-30c-1 (F), exo-miR-186 (G), exo-miR-199b (H), exo-miR-200b (I), exo-miR-330 (J), exo-miR-335 (K), exo-miR-346 (L), exo-miR-446 (M), exo-miR-574 (N), exo-miR-3126 (O). Data are presented as MA plots (A-1~O-1) and cumulative distributions (A-2~O-2) of seed-matched target genes of miR-24-3p.

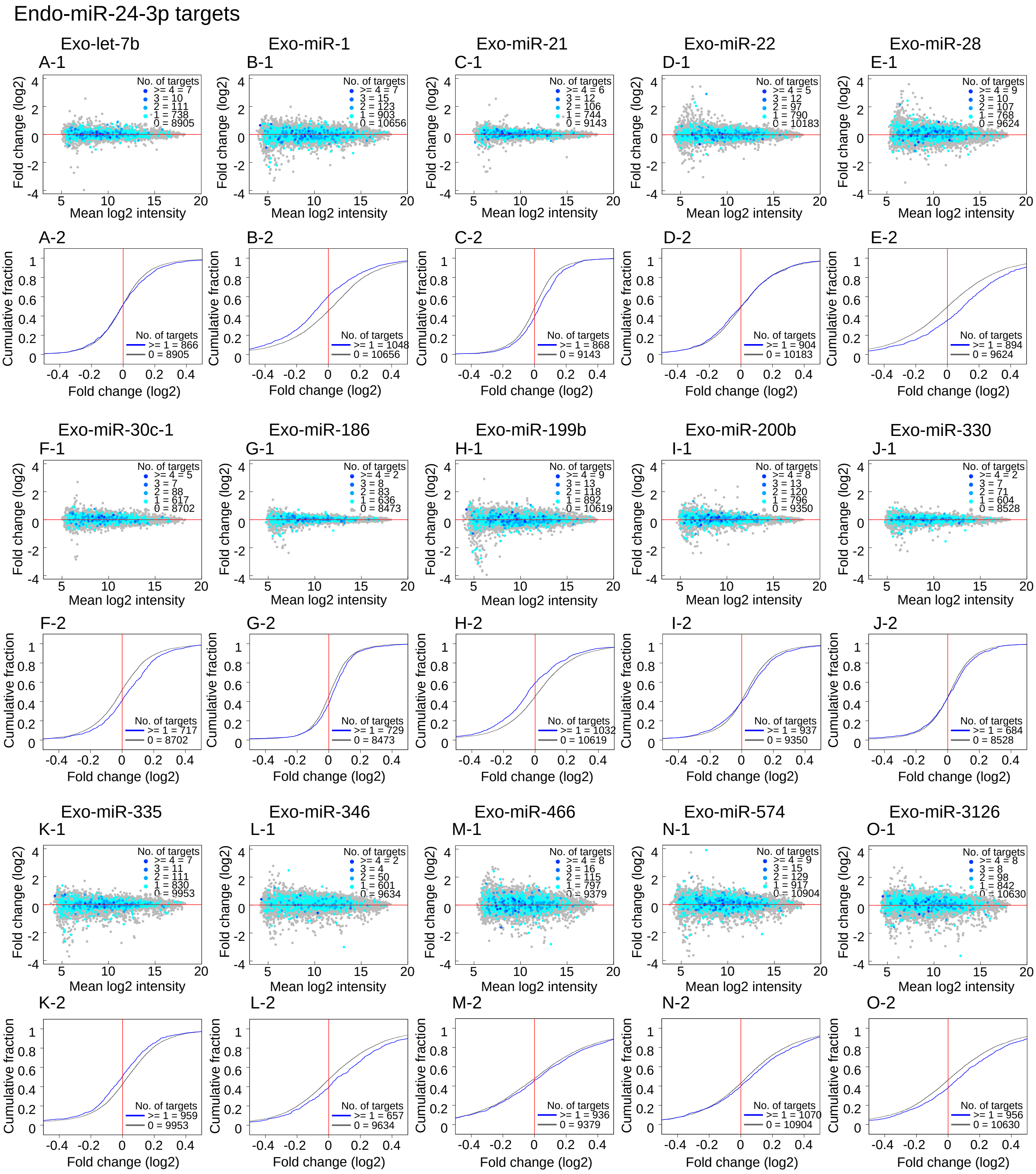

Figure S8. Microarray analysis of the expression of target genes of the endo-miRNA, miR-30a-5p Microarray analysis of endo-miR-30a-5p target gene expression in cells transfected with exo-let-7b (A), exo-miR-1 (B), exo-miR-21 (C), exo-miR-22 (D), exo-miR-28 (E), exo-miR-30c-1 (F), exo-miR-186 (G), exo-miR-199b (H), exo-miR-200b (I), exo-miR-330 (J), exo-miR-335 (K), exo-miR-346 (L), exo-miR-446 (M), exo-miR-574 (N), exo-miR-3126 (O). Data are presented as MA plots (A-1~O-1) and cumulative distributions (A-2~O-2) of seed-matched target genes of miR-30a-5p.

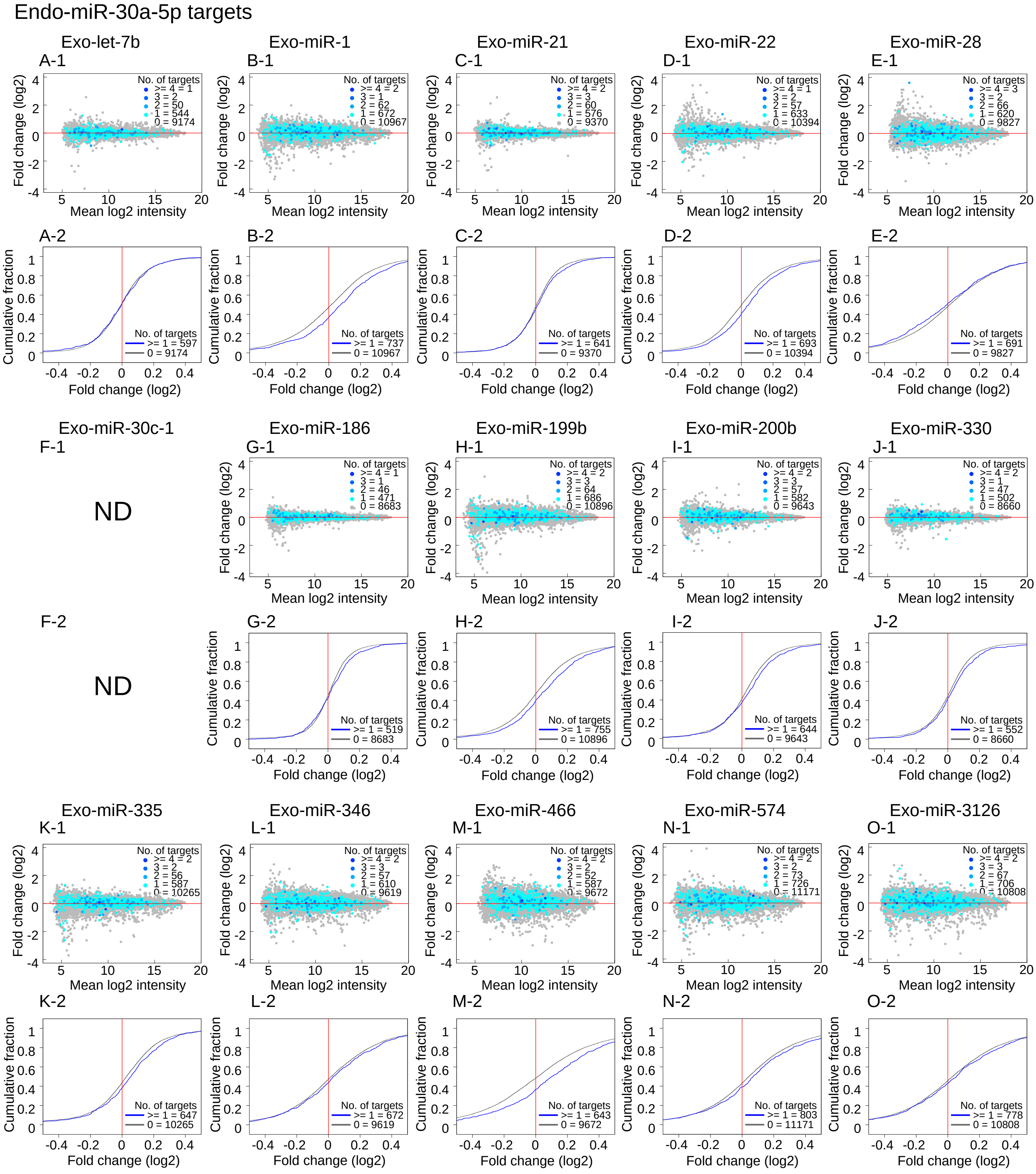

Figure S9. Microarray analysis of the expression of target genes of the endo-miRNA, miR-92a-3p Microarray analysis of endo-miR-92a-3p target gene expression in cells transfected with exo-let-7b (A), exo-miR-1 (B), exo-miR-21 (C), exo-miR-22 (D), exo-miR-28 (E), exo-miR-30c-1 (F), exo-miR-186 (G), exo-miR-199b (H), exo-miR-200b (I), exo-miR-330 (J), exo-miR-335 (K), exo-miR-346 (L), exo-miR-446 (M), exo-miR-574 (N), exo-miR-3126 (O). Data are presented as MA plots (A-1~O-1) and cumulative distributions (A-2~O-2) of seed-matched target genes of miR-92a-3p.

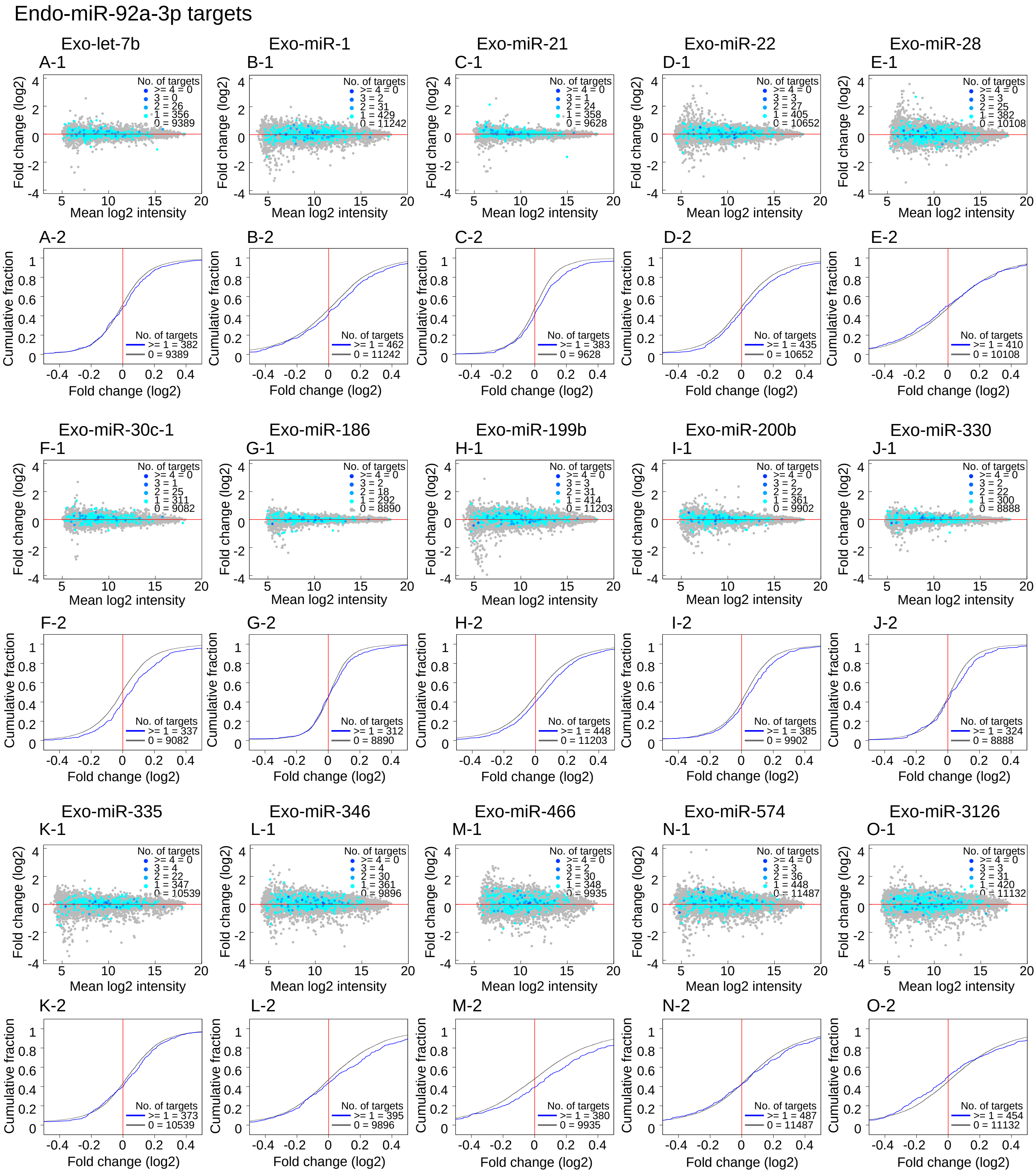

Figure S10. Microarray analysis of the expression of target genes of the endo-miRNA, miR-19a-3p Microarray analysis of endo-miR-19a-3p target gene expression in cells transfected with exo-let-7b (A), exo-miR-1 (B), exo-miR-21 (C), exo-miR-22 (D), exo-miR-28 (E), exo-miR-30c-1 (F), exo-miR-186 (G), exo-miR-199b (H), exo-miR-200b (I), exo-miR-330 (J), exo-miR-335 (K), exo-miR-346 (L), exo-miR-446 (M), exo-miR-574 (N), exo-miR-3126 (O). Data are presented as MA plots (A-1~O-1) and cumulative distributions (A-2~O-2) of seed-matched target genes of miR-19a-3p.

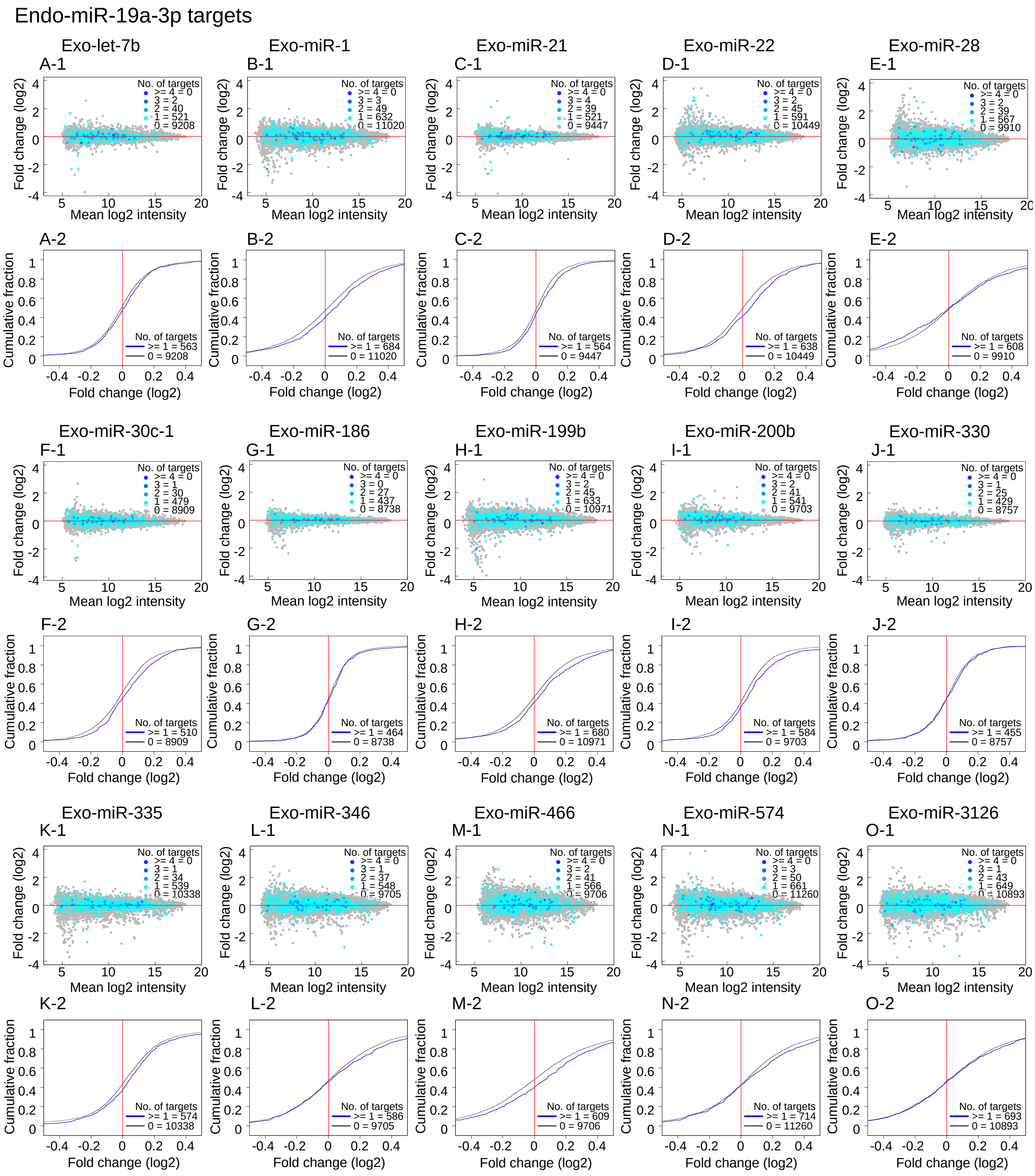

Figure S11. Microarray analysis of the expression of target genes of the endo-miRNA, miR-15a-5p Microarray analysis of endo-miR-15a-5p target gene expression in cells transfected with exo-let-7b (A), exo-miR-1 (B), exo-miR-21 (C), exo-miR-22 (D), exo-miR-28 (E), exo-miR-30c-1 (F), exo-miR-186 (G), exo-miR-199b (H), exo-miR-200b (I), exo-miR-330 (J), exo-miR-335 (K), exo-miR-346 (L), exo-miR-446 (M), exo-miR-574 (N), exo-miR-3126 (O). Data are presented as MA plots (A-1~O-1) and cumulative distributions (A-2~O-2) of seed-matched target genes of miR-15a-5p.

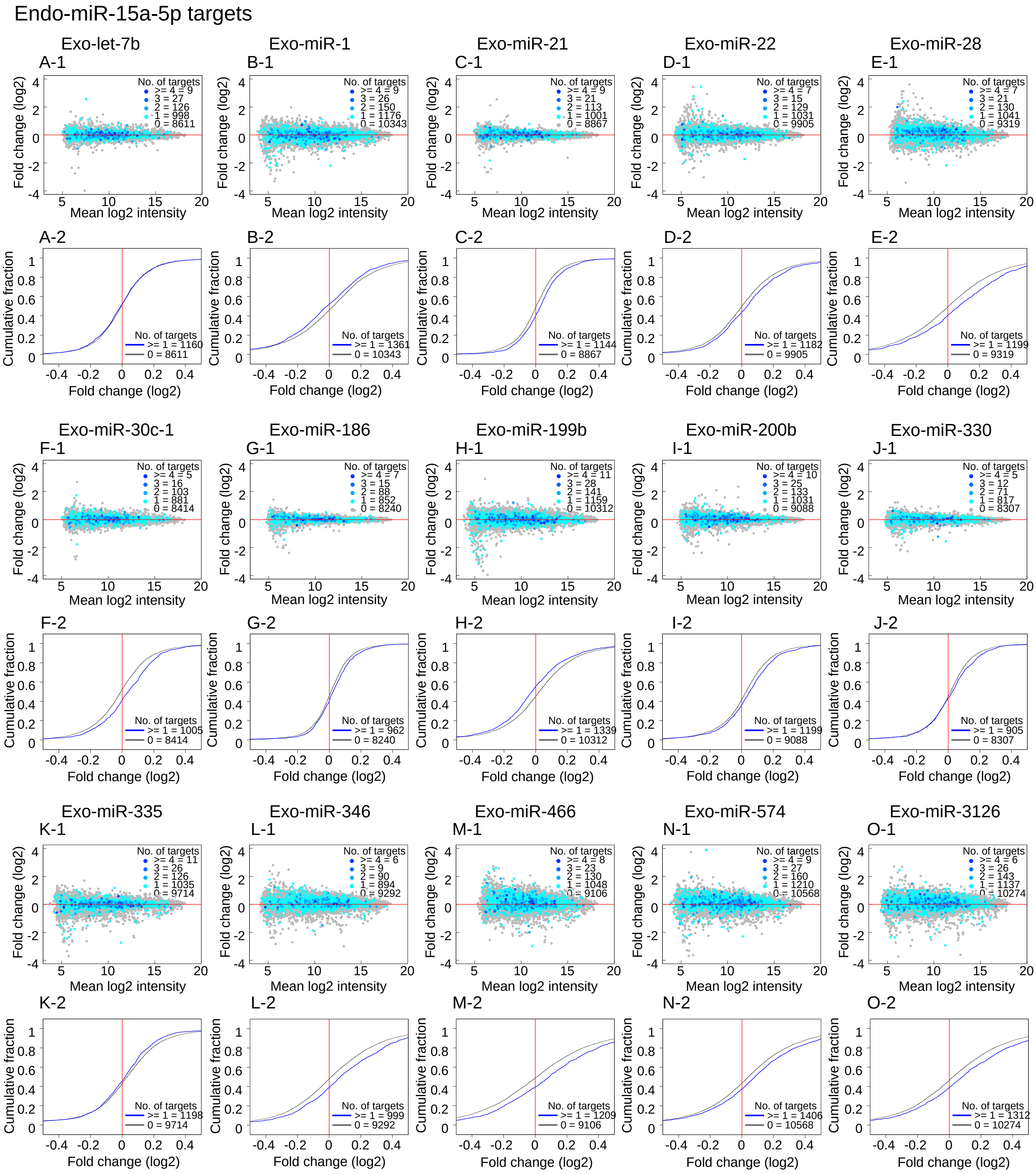

Figure S12. Microarray analysis of the expression of target genes of the endo-miRNA, miR-22-3p Microarray analysis of endo-miR-22-3p target gene expression in cells transfected with exo-let-7b (A), exo-miR-1 (B), exo-miR-21 (C), exo-miR-22 (D), exo-miR-28 (E), exo-miR-30c-1 (F), exo-miR-186 (G), exo-miR-199b (H), exo-miR-200b (I), exo-miR-330 (J), exo-miR-335 (K), exo-miR-346 (L), exo-miR-446 (M), exo-miR-574 (N), exo-miR-3126 (O). Data are presented as MA plots (A-1~O-1) and cumulative distributions (A-2~O-2) of seed-matched target genes of miR-22-3p.

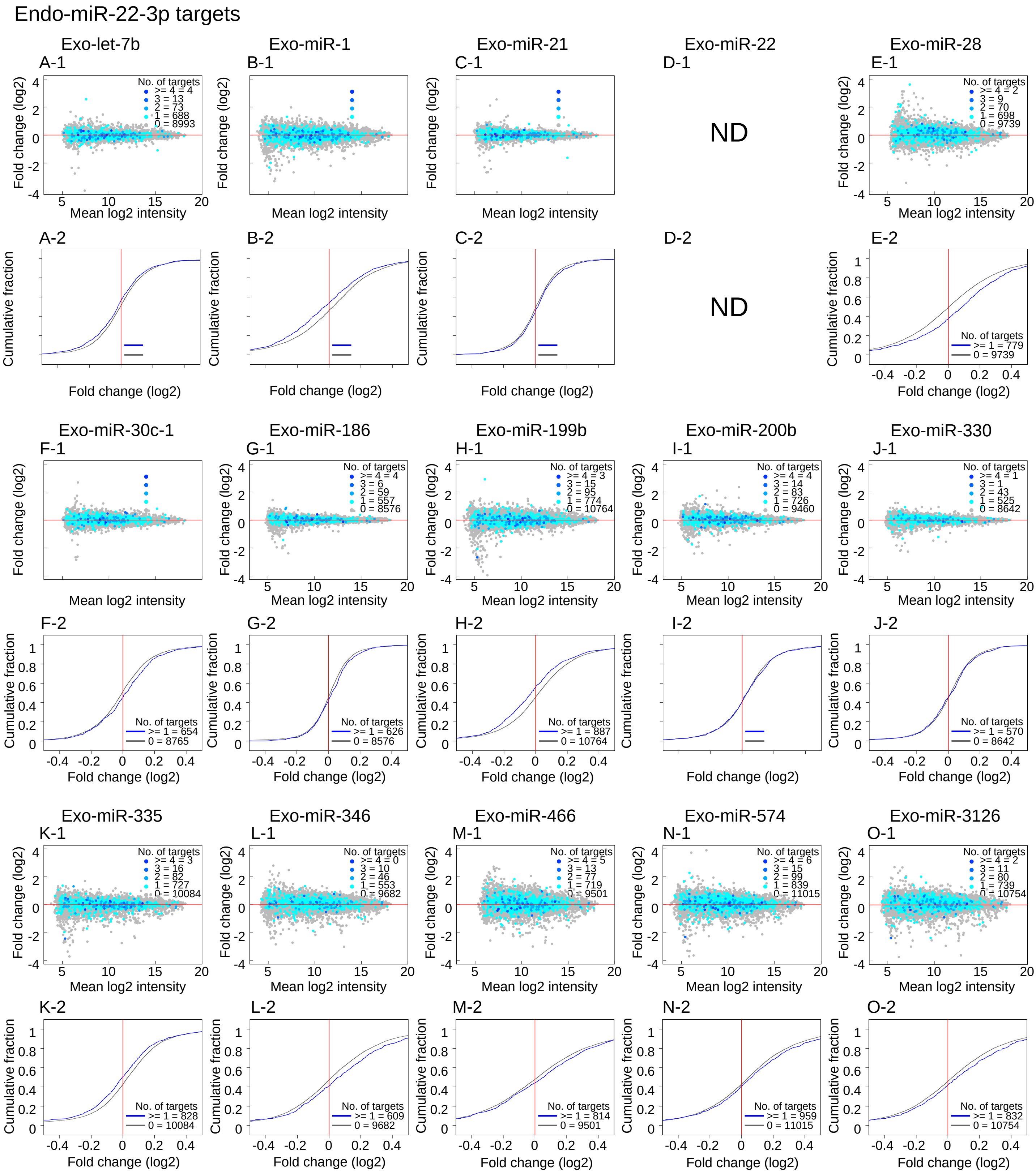

Figure S13. Microarray analysis of the expression of target genes of the endo-miRNA, miR-29a-3p Microarray analysis of endo-miR-29a-3p target gene expression in cells transfected with exo-let-7b (A), exo-miR-1 (B), exo-miR-21 (C), exo-miR-22 (D), exo-miR-28 (E), exo-miR-30c-1 (F), exo-miR-186 (G), exo-miR-199b (H), exo-miR-200b (I), exo-miR-330 (J), exo-miR-335 (K), exo-miR-346 (L), exo-miR-446 (M), exo-miR-574 (N), exo-miR-3126 (O). Data are presented as MA plots (A-1~O-1) and cumulative distributions (A-2~O-2) of seed-matched target genes of miR-29a-3p.

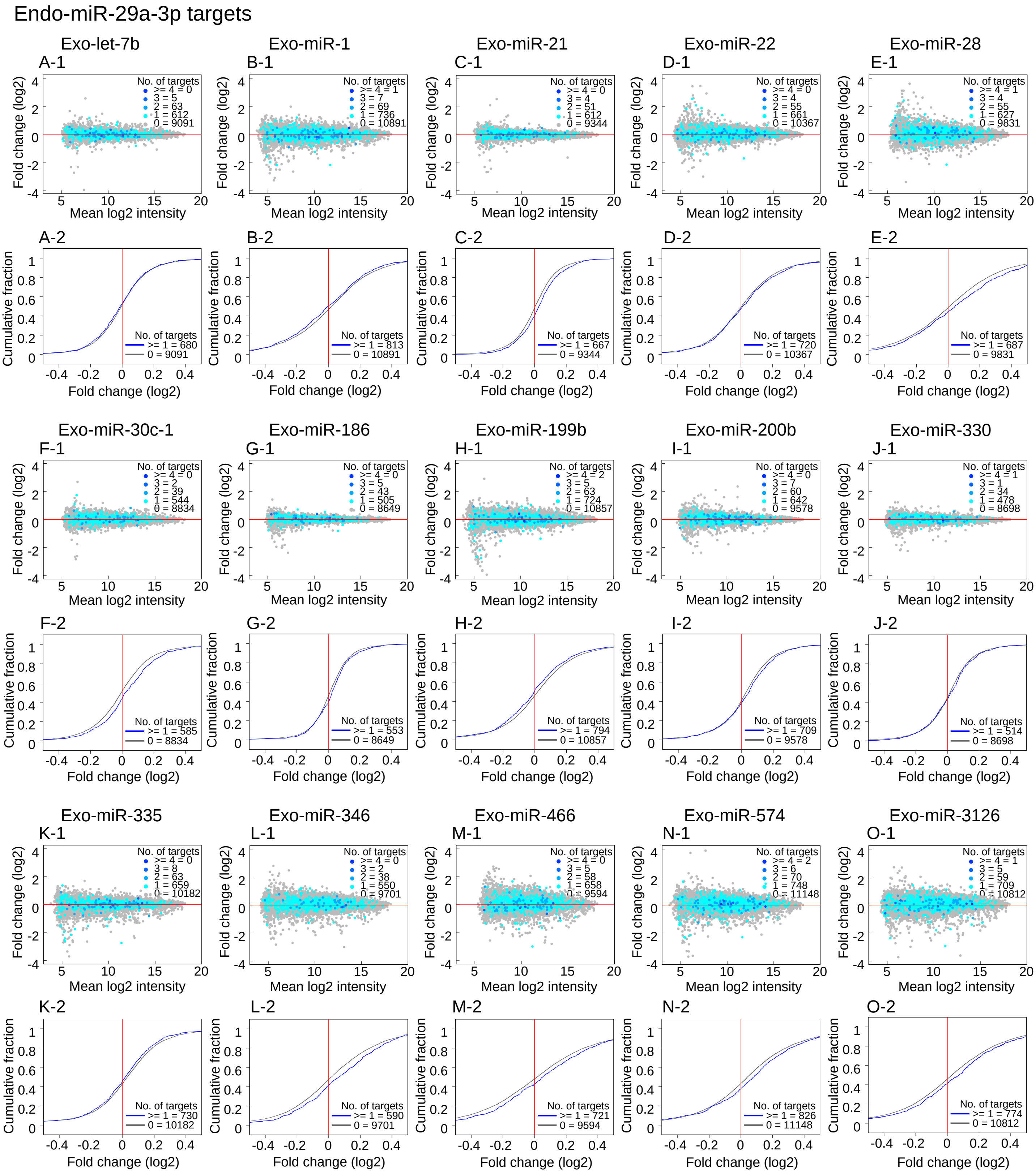

Figure S14. Microarray analysis of the expression of target genes of the endo-miRNA, miR-125a-5p Microarray analysis of endo-miR-125a-5p target gene expression in cells transfected with exo-let-7b (A), exo-miR-1 (B), exo-miR-21 (C), exo-miR-22 (D), exo-miR-28 (E), exo-miR-30c-1 (F), exo-miR-186 (G), exo-miR-199b (H), exo-miR-200b (I), exo-miR-330 (J), exo-miR-335 (K), exo-miR-346 (L), exo-miR-446 (M), exo-miR-574 (N), exo-miR-3126 (O). Data are presented as MA plots (A-1~O-1) and cumulative distributions (A-2~O-2) of seed-matched target genes of miR-125a-5p.

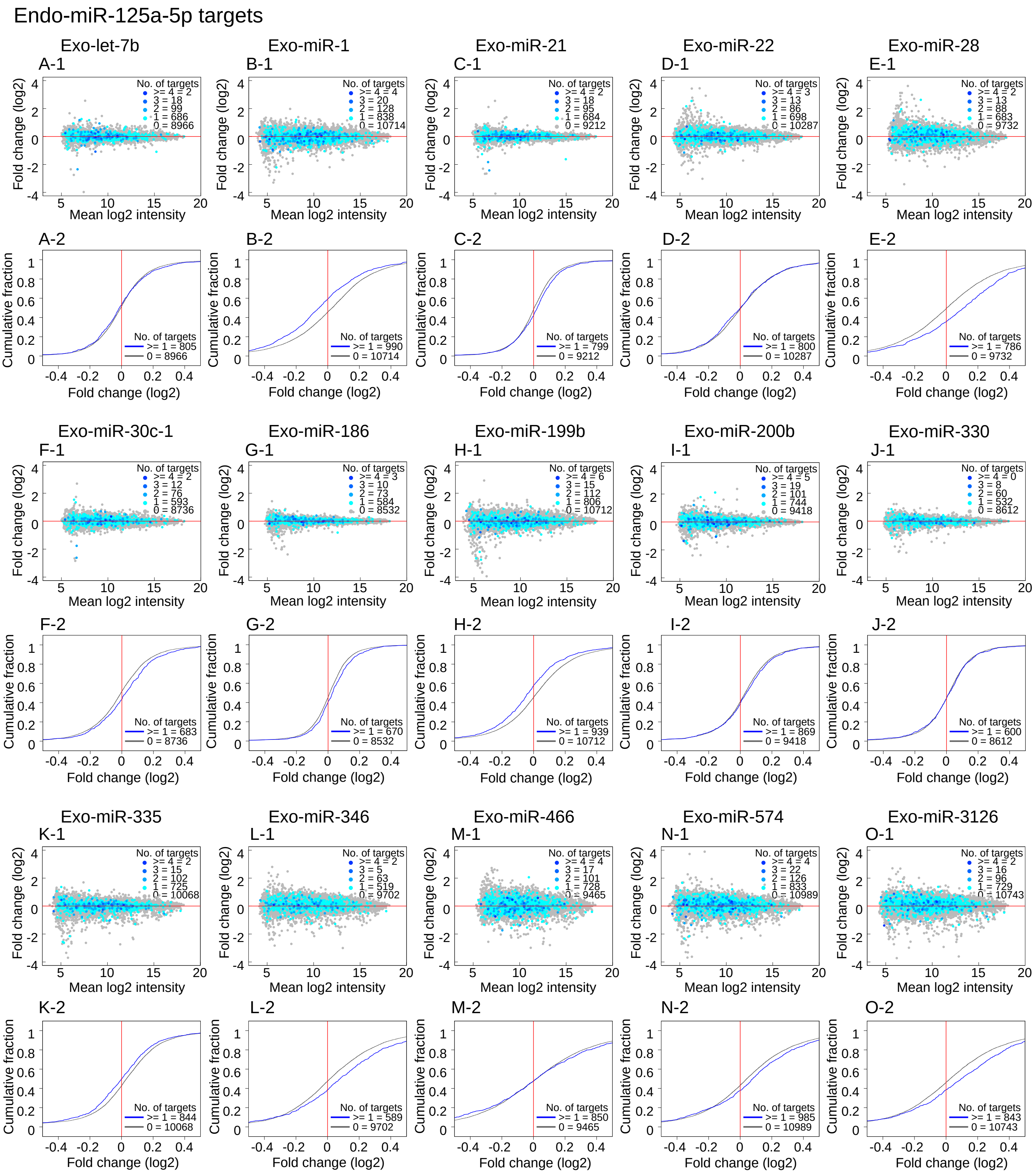

Figure S15. Microarray analysis of the expression of target genes of the endo-miRNA, miR-93-5p Microarray analysis of endo-miR-93-5p target gene expression in cells transfected with exo-let-7b (A), exo-miR-1 (B), exo-miR-21 (C), exo-miR-22 (D), exo-miR-28 (E), exo-miR-30c-1 (F), exo-miR-186 (G), exo-miR-199b (H), exo-miR-200b (I), exo-miR-330 (J), exo-miR-335 (K), exo-miR-346 (L), exo-miR-446 (M), exo-miR-574 (N), exo-miR-3126 (O). Data are presented as MA plots (A-1~O-1) and cumulative distributions (A-2~O-2) of seed-matched target genes of miR-93-5p.

Endo-miR-93-5p targets

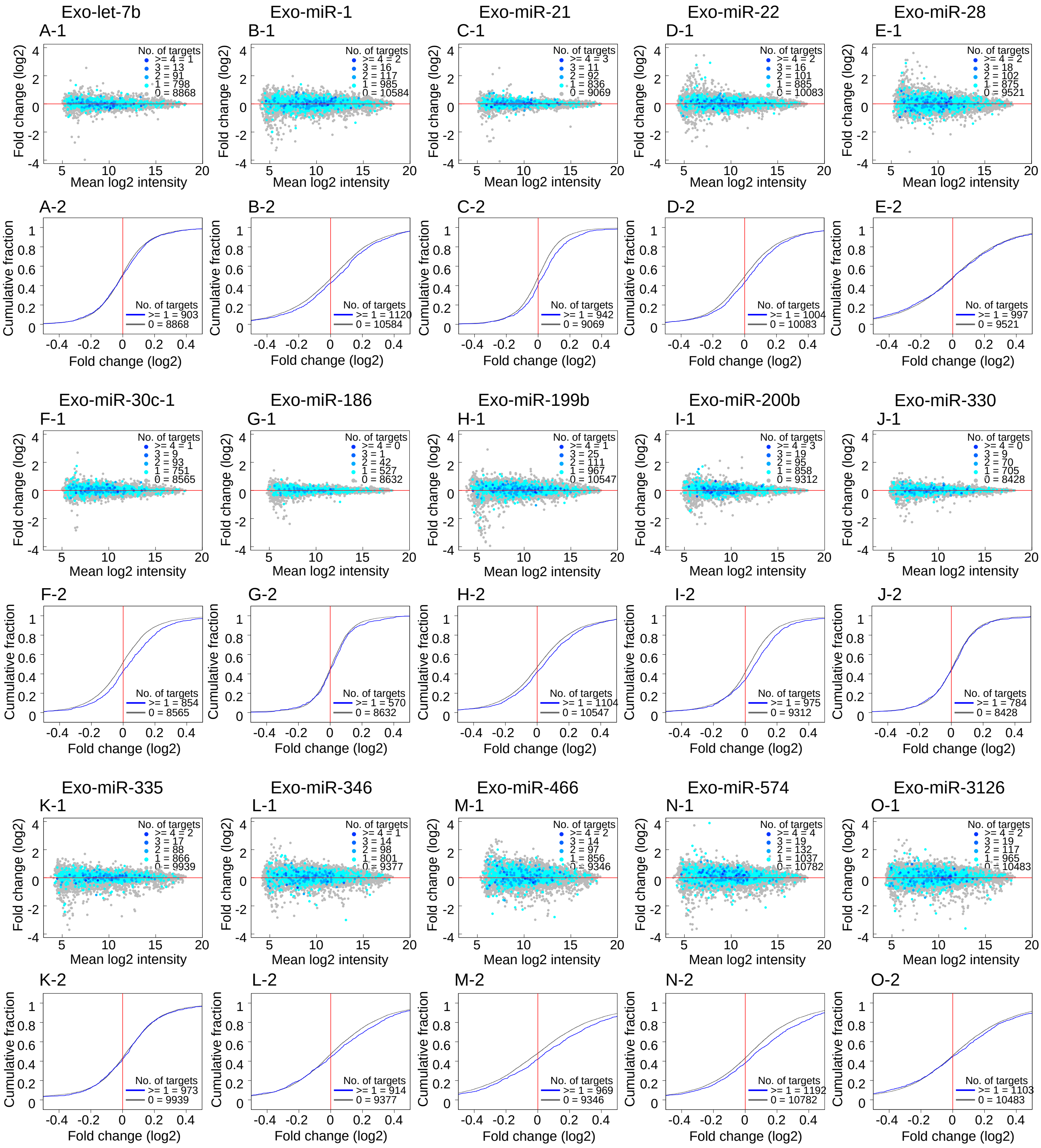

Figure S16. Microarray analysis of the expression of target genes of the endo-miRNA, miR-191-5p Microarray analysis of endo-miR-191-5p target gene expression in cells transfected with exo-let-7b (A), exo-miR-1 (B), exo-miR-21 (C), exo-miR-22 (D), exo-miR-28 (E), exo-miR-30c-1 (F), exo-miR-186 (G), exo-miR-199b (H), exo-miR-200b (I), exo-miR-330 (J), exo-miR-335 (K), exo-miR-346 (L), exo-miR-446 (M), exo-miR-574 (N), exo-miR-3126 (O). Data are presented as MA plots (A-1~O-1) and cumulative distributions (A-2~O-2) of seed-matched target genes of miR-191-5p.

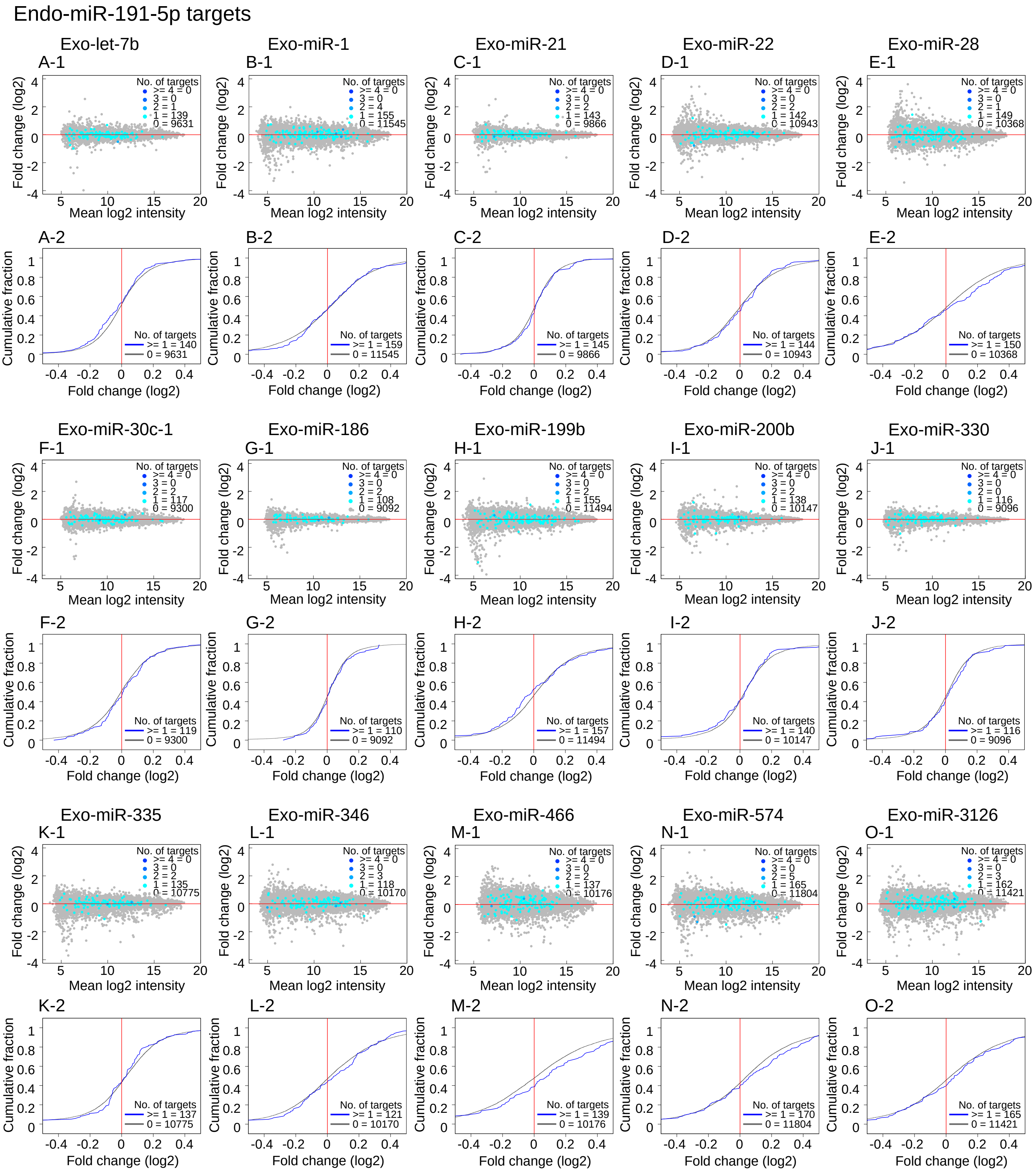

Figure S17. Microarray analysis of the expression of target genes of the endo-miRNA, miR-103a-3p Microarray analysis of endo-miR-103a-3p target gene expression in cells transfected with exo-let-7b (A), exo-miR-1 (B), exo-miR-21 (C), exo-miR-22 (D), exo-miR-28 (E), exo-miR-30c-1 (F), exo-miR-186 (G), exo-miR-199b (H), exo-miR-200b (I), exo-miR-330 (J), exo-miR-335 (K), exo-miR-346 (L), exo-miR-446 (M), exo-miR-574 (N), exo-miR-3126 (O). Data are presented as MA plots (A-1~O-1) and cumulative distributions (A-2~O-2) of seed-matched target genes of miR-103a-3p.

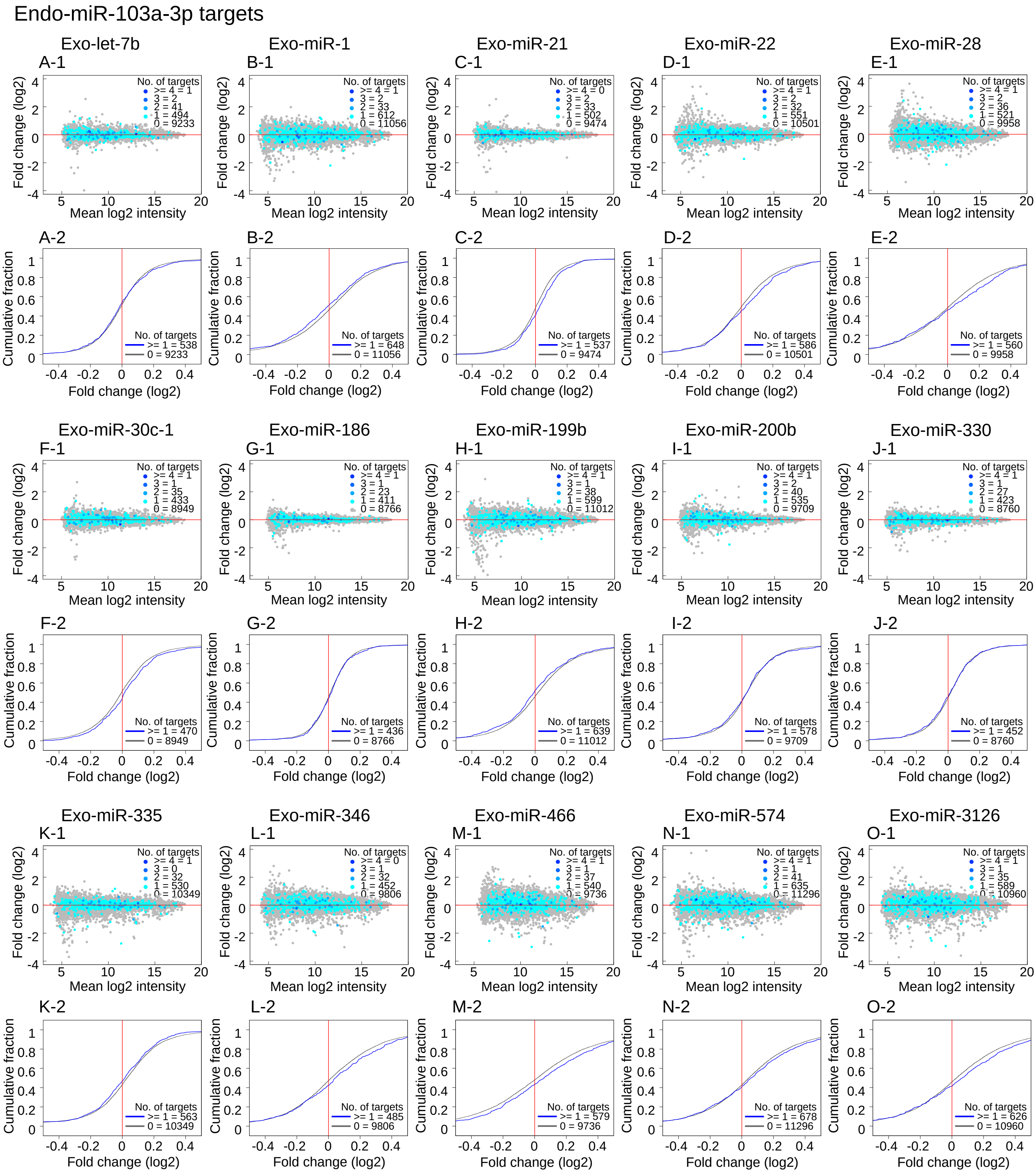

Figure S18. Microarray analysis of the expression of target genes of the endo-miRNA, miR-143-3p Microarray analysis of endo-miR-143-3p target gene expression in cells transfected with exo-let-7b (A), exo-miR-1 (B), exo-miR-21 (C), exo-miR-22 (D), exo-miR-28 (E), exo-miR-30c-1 (F), exo-miR-186 (G), exo-miR-199b (H), exo-miR-200b (I), exo-miR-330 (J), exo-miR-335 (K), exo-miR-346 (L), exo-miR-446 (M), exo-miR-574 (N), exo-miR-3126 (O). Data are presented as MA plots (A-1~O-1) and cumulative distributions (A-2~O-2) of seed-matched target genes of miR-143-3p.

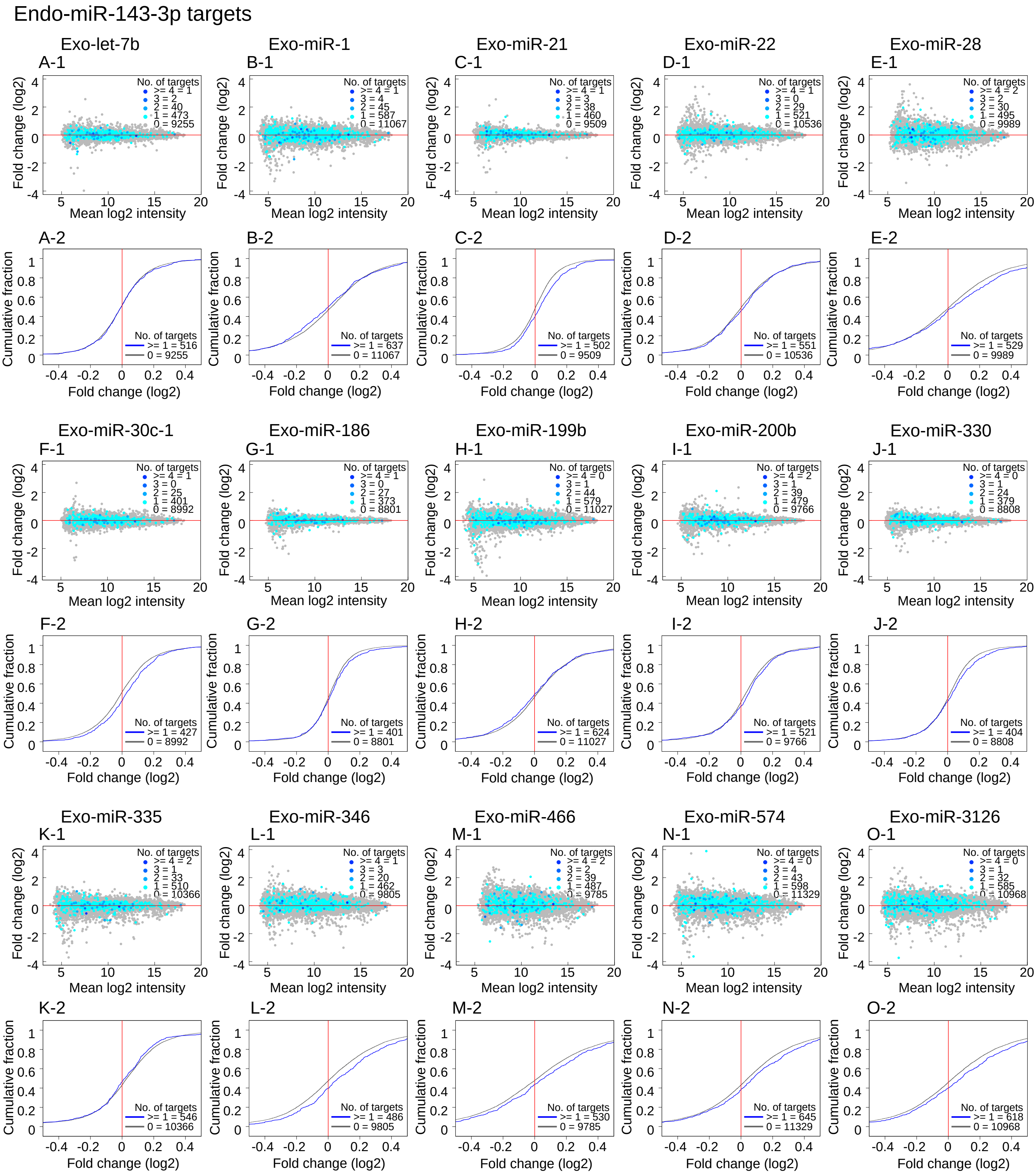

Figure S19. Microarray analysis of the expression of target genes of the endo-miRNA, miR-100-5p Microarray analysis of endo-miR-100-5p target gene expression in cells transfected with exo-let-7b (A), exo-miR-1 (B), exo-miR-21 (C), exo-miR-22 (D), exo-miR-28 (E), exo-miR-30c-1 (F), exo-miR-186 (G), exo-miR-199b (H), exo-miR-200b (I), exo-miR-330 (J), exo-miR-335 (K), exo-miR-346 (L), exo-miR-446 (M), exo-miR-574 (N), exo-miR-3126 (O). Data are presented as MA plots (A-1~O-1) and cumulative distributions (A-2~O-2) of seed-matched target genes of miR-100-5p.

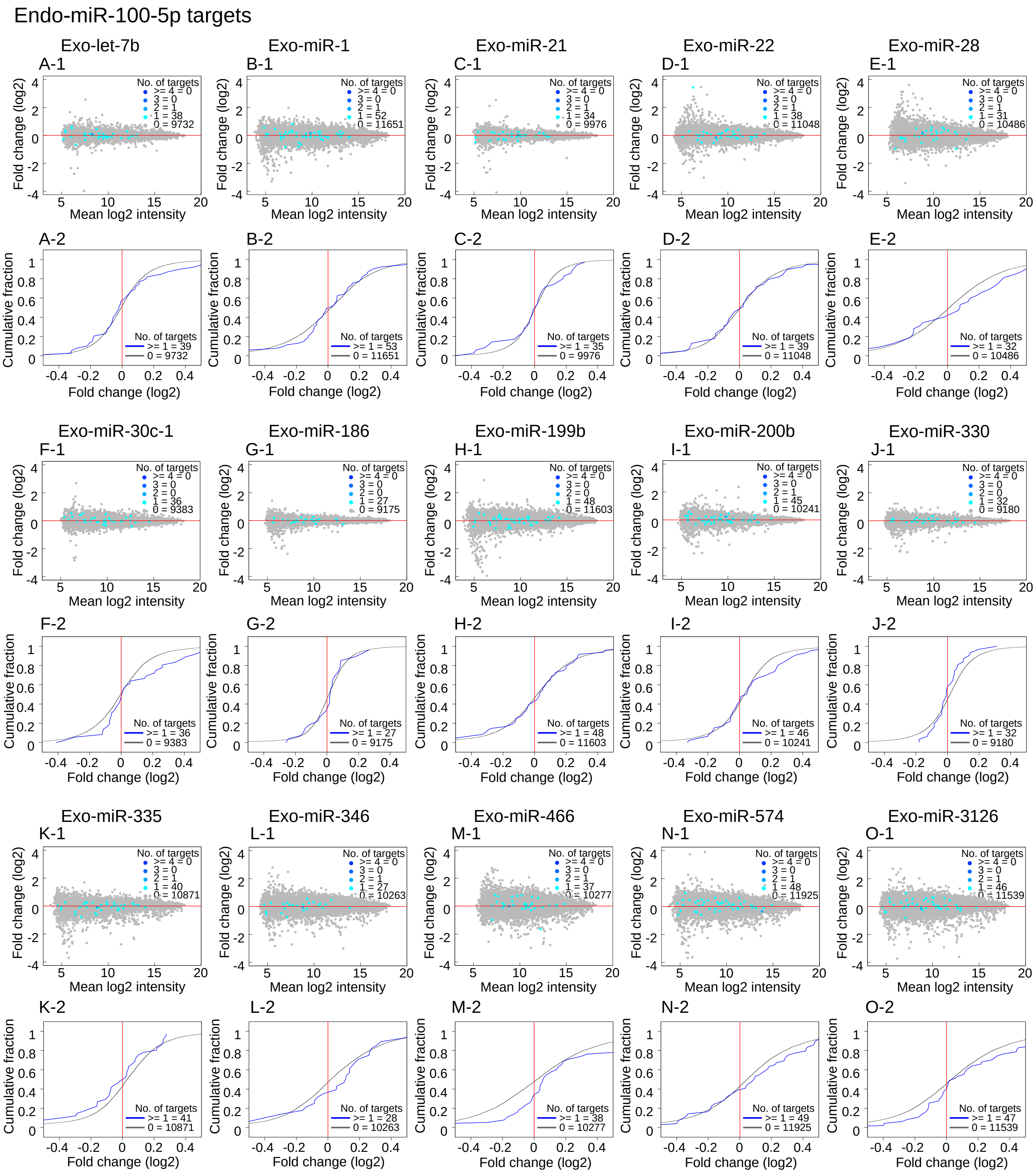

Figure S20. Microarray analysis of the expression of target genes of the endo-miRNA, miR-23a-3p Microarray analysis of endo-miR-23a-3p target gene expression in cells transfected with exo-let-7b (A), exo-miR-1 (B), exo-miR-21 (C), exo-miR-22 (D), exo-miR-28 (E), exo-miR-30c-1 (F), exo-miR-186 (G), exo-miR-199b (H), exo-miR-200b (I), exo-miR-330 (J), exo-miR-335 (K), exo-miR-346 (L), exo-miR-446 (M), exo-miR-574 (N), exo-miR-3126 (O). Data are presented as MA plots (A-1~O-1) and cumulative distributions (A-2~O-2) of seed-matched target genes of miR-23a-3p.

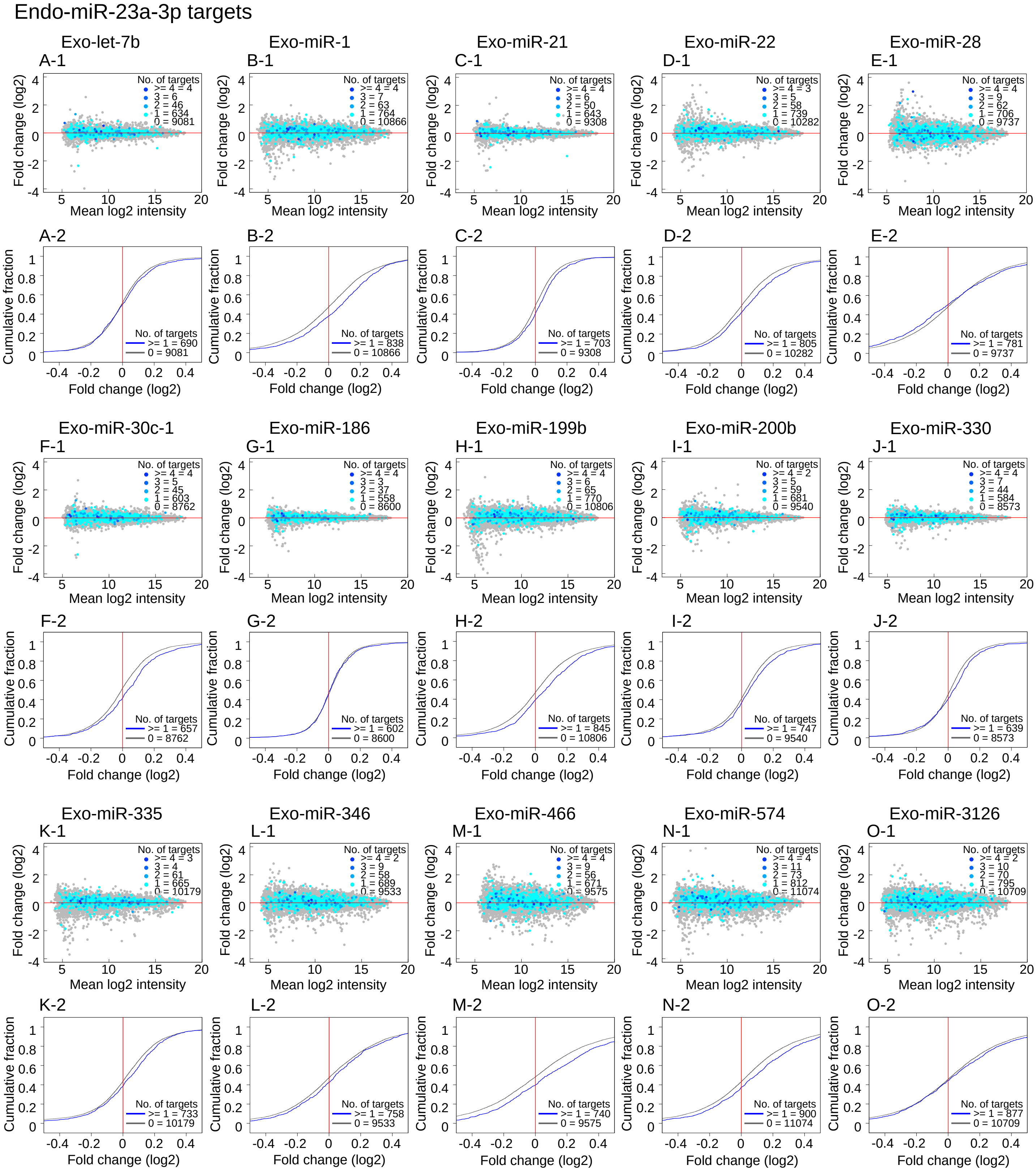

Figure S21. Microarray analysis of the expression of target genes of the endo-miRNA, miR-186-5p Microarray analysis of endo-miR-186-5p target gene expression in cells transfected with exo-let-7b (A), exo-miR-1 (B), exo-miR-21 (C), exo-miR-22 (D), exo-miR-28 (E), exo-miR-30c-1 (F), exo-miR-186 (G), exo-miR-199b (H), exo-miR-200b (I), exo-miR-330 (J), exo-miR-335 (K), exo-miR-346 (L), exo-miR-446 (M), exo-miR-574 (N), exo-miR-3126 (O). Data are presented as MA plots (A-1~O-1) and cumulative distributions (A-2~O-2) of seed-matched target genes of miR-186-5p.

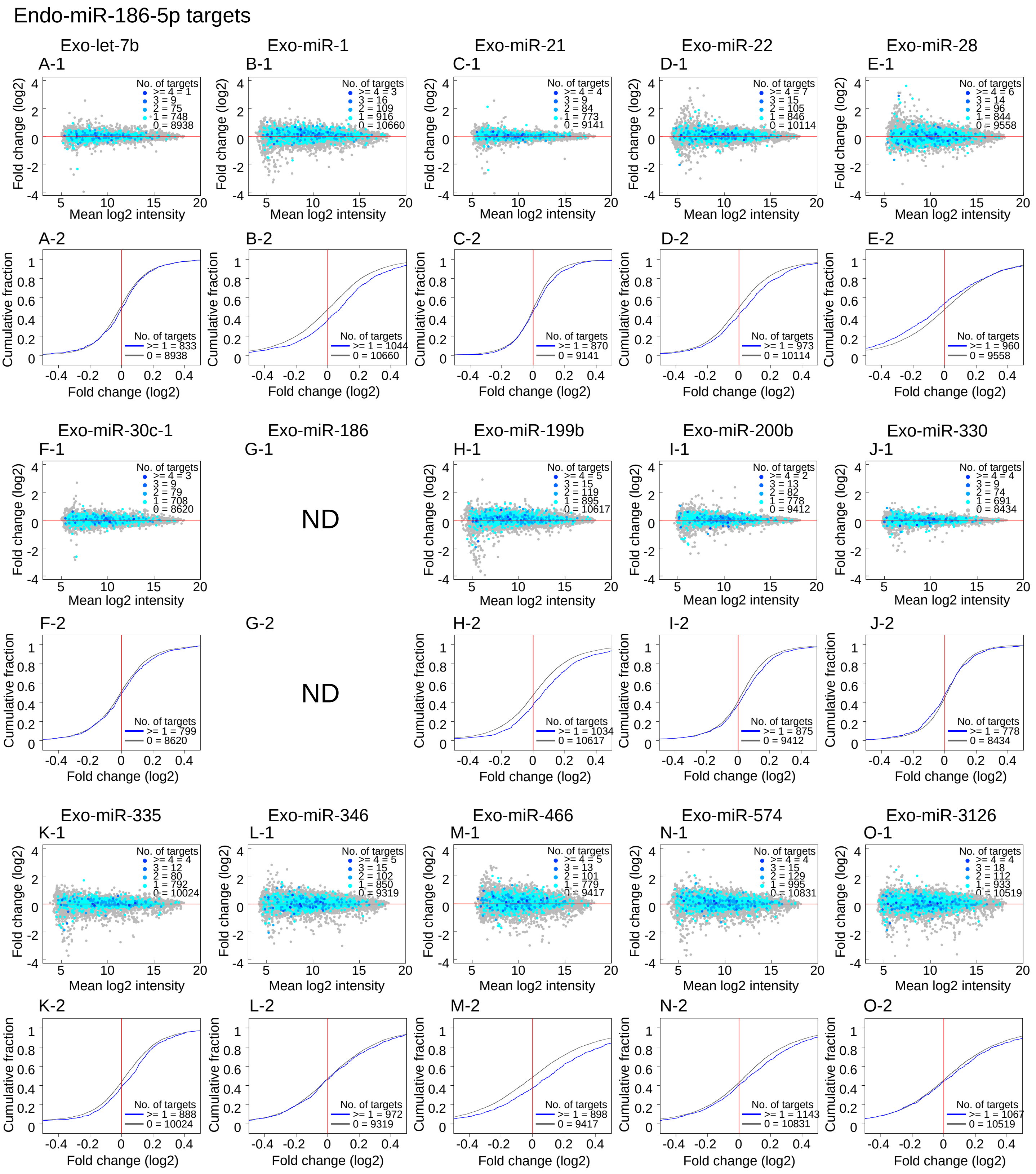

Figure S22. Mean fold changes of the expression levels of endo-miRNA target transcripts by the transfection of different exo-miRNAs. HeLa cells were transfected with each of fifteen exo-miRNA duplexes, $\log_2$ mean differential fold changes of the expression levels of seed-matched target genes of each of top 20 endo-miRNAs were calculated. Data were shown with respect to endo-miRNAs, let-7b-5p (**A**), miR-21 (**B**), mR-27a-3p (**C**), miR-17-5p (**D**), miR-26a-5p (**E**), miR-24-3p (**F**), miR-30a-5p (**G**), miR-92a-5p (**H**), miR-19a-5p (**I**), miR-15a-5p (**J**), miR-22-3p (**K**), miR-29a-3p (**L**), miR-125a-5p (**M**), miR-93-5p (**N**), miR-191-5p (**O**), miR-103a-3p (**P**), miR-143-3p (**Q**), miR-100-5p (**R**), miR-23a-3p (**S**), and mR-186-5p (**T**), and their averaged values were shown in (**U**). Note that each exo-miRNA increased the endo-miRNA target genes in different degrees. The individual data are shown in Figures S2-S21. Data represent the mean ± S.E (*, $P < 0.05$).

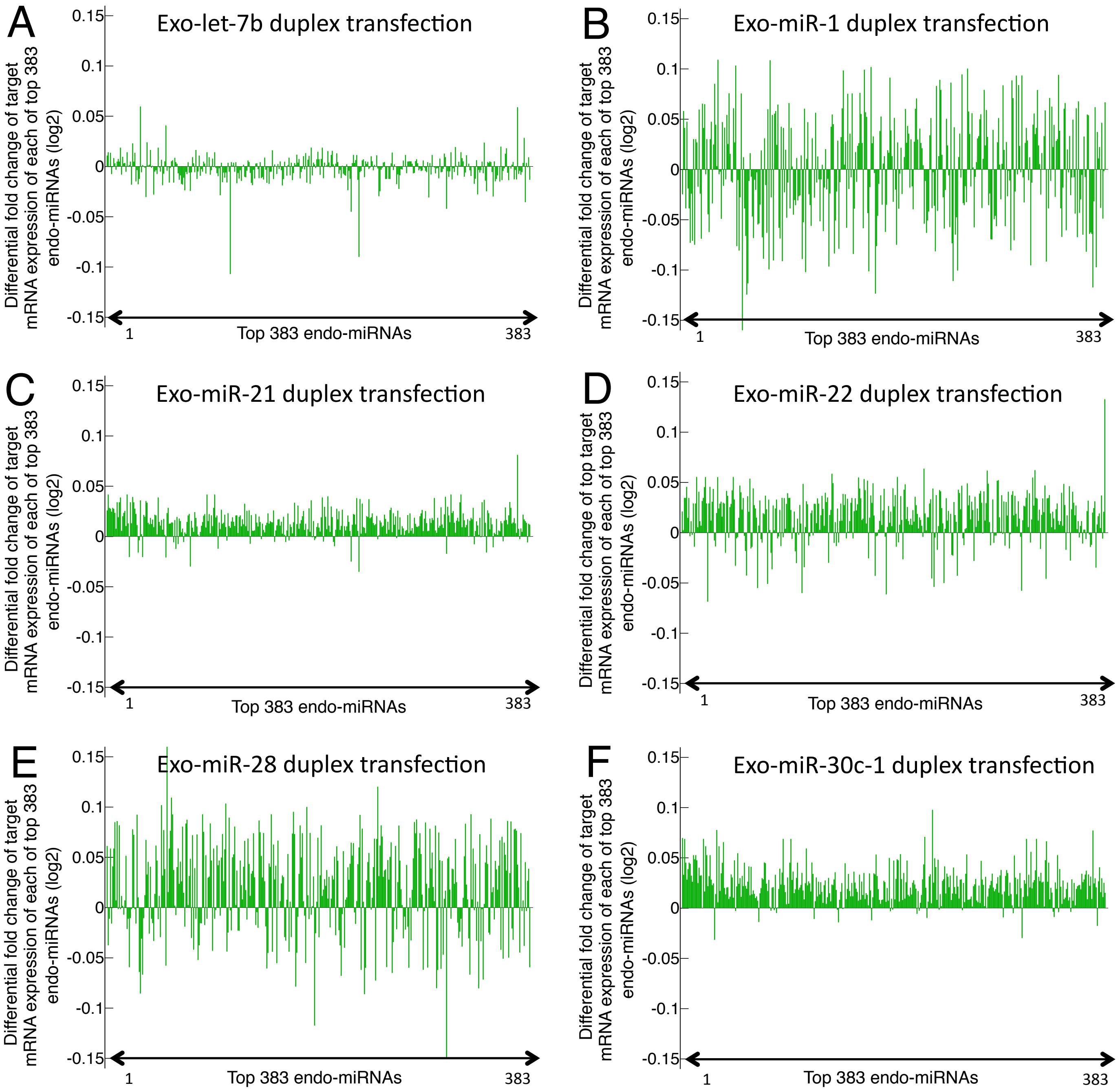

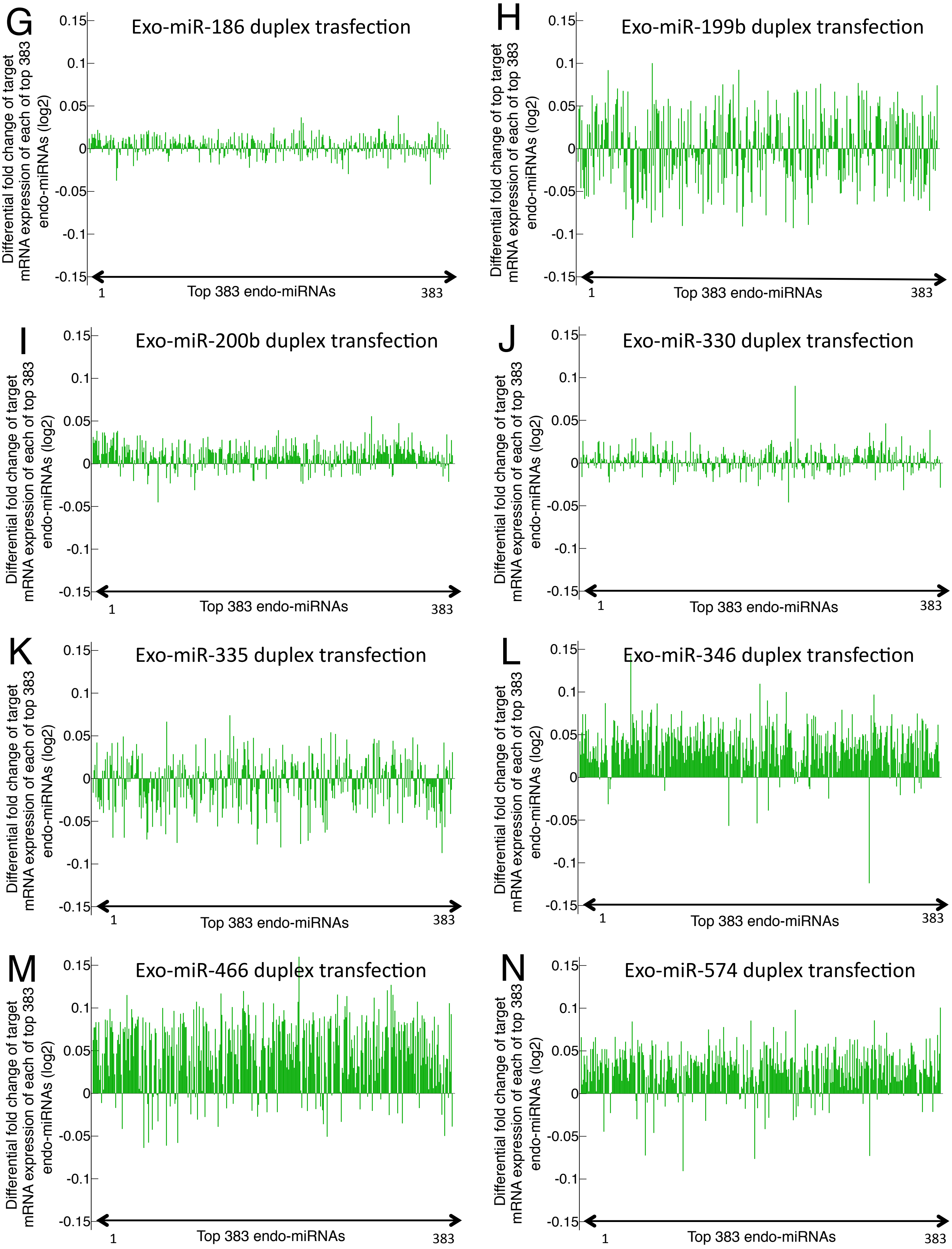
G
Exo-miR-186 duplex trasfection
H
Exo-miR-199b duplex transfection
I
Exo-miR-200b duplex transfection
J
Exo-miR-330 duplex transfection
K
Exo-miR-335 duplex transfection
L
Exo-miR-346 duplex transfection
M
Exo-miR-466 duplex transfection
N
Exo-miR-574 duplex transfection
Differential fold change of target mRNA expression of each of top 383 endo-miRNAs (log2)
Differential fold change of top target mRNA expression of each of top 383 endo-miRNAs (log2)
0.15
0.1
0.05
0
-0.05
-0.1
-0.15
1
Top 383 endo-miRNAs
383

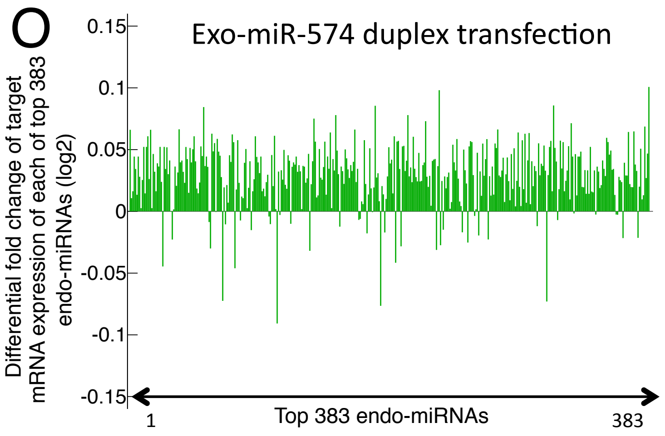
O
Exo-miR-574 duplex transfection
Differential fold change of target mRNA expression of each of top 383 endo-miRNAs (log2)
0.15
0.1
0.05
0
-0.05
-0.1
-0.15
1
Top 383 endo-miRNAs
383

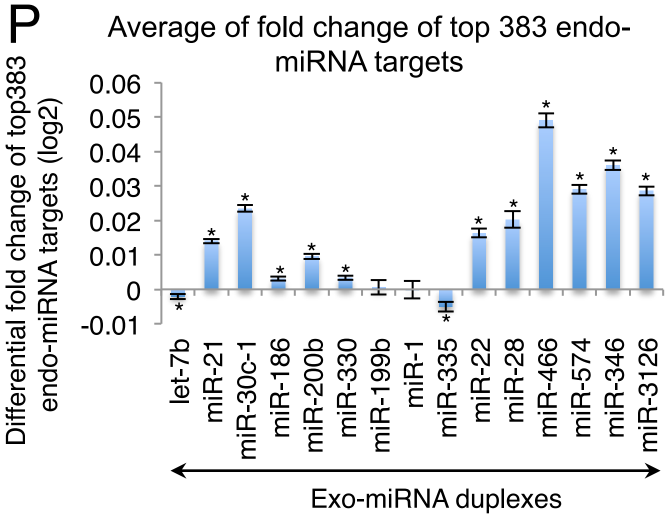
P
Average of fold change of top 383 endo-miRNA targets
Differential fold change of top383 endo-miRNA targets (log2)
0.06
0.05
0.04
0.03
0.02
0.01
0
-0.01
let-7b
miR-21
miR-30c-1
miR-186
miR-200b
miR-330
miR-199b
miR-1
miR-335
miR-22
miR-28
miR-466
miR-574
miR-346
miR-3126
Exo-miRNA duplexes

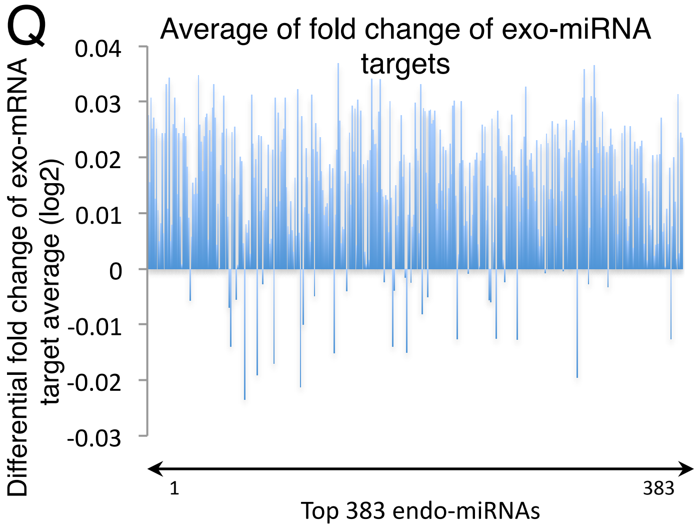
Q
Average of fold change of exo-miRNA targets
Differential fold change of exo-miRNA target average (log2)
0.04
0.03
0.02
0.01
0
-0.01
-0.02
-0.03
1
383
Top 383 endo-miRNAs

Figure S23. Confirmation of microarray data by quantitative RT-PCR. A total of 28genes were subjected to quantitative analysis, and the results were compared with the corresponding microarray data. HeLa cells were transfected with miRNA duplexes, let-7b, miR-21, miR-30c-1, miR-186, miR-200b, and the expressions of the following were measured by quantitative RT-PCR: let-7b-5p targets, HDHD1A, UBQLN, PBX2, and ZC3H11A; miR-21-5p targets, SKA2, CASP7, SLC11A2, and UBR3; miR-30c-5p targets, RARG, SPAST, METTL7A, and SEC23A; miR-186-5p targets, ZNF652, SLC39A1, OTUD4, and USP37; miR-200b-5p targets, AP1S2, OSTM1, SHCBP1, and PGM2L1. The correlation coefficient between the quantitative RT-PCR and microarray expression data was 0.74.

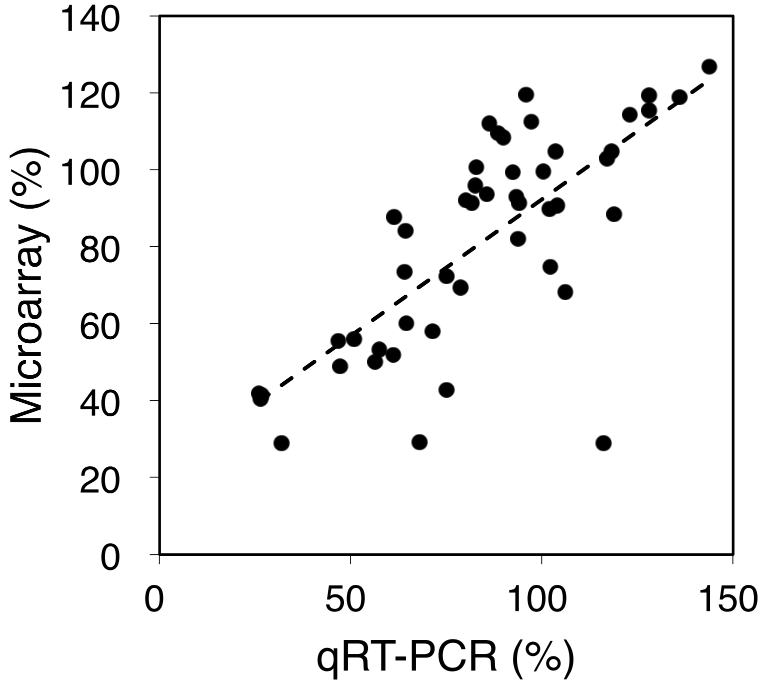

Supplement: Supplementary file 1 [file ijms-14-11171-s001.pdf]
