# Supplementary material for: Fluctuation of Global Gene Expression by Endogenous miRNA Response to the Introduction of an Exogenous miRNA
Source: Int J Mol Sci. 2013 May 27;14(6):11171–89. doi: 10.3390/ijms140611171 (PMC3709726; doi:10.3390/ijms140611171)
Supplement: Supplementary file 2 [file ijms-14-11171-s002.pdf]

Supplementary Information

Table S1. Synthesized oligonucleotides for miRNA duplexes.

Duplex	One strand	Sequence *	Opposite strand	Sequence *
**let-7b**	let-7b-5p	UGAGGUAGUAGGUUGUGUGGUU	let-7b-3p	CUAUACAACCUACUGCCUUCCC
**miR-1**	miR-1-5p	UGGAAUGUAAAGAAGUAUGUAU	miR-3p	ACAUACUUCUUUAUAUGCCCAUA
**miR-21**	miR-21-5p	UAGCUUAUCAGACUGAUGUUGA	miR-21-3p	CAACACCAGUCGAUGGGCUGU
**miR-22**	miR-22-3p	AAGCUGCCAGUUGAAGAACUGU	miR-22-5p	AGUUCUUCAGUGGCAAGCUUUA
**miR-28**	miR-28-5p	AAGGAGCUCACAGUCUAUUGAG	miR-28-3p	CACUAGAUUGUGAGCUCCUGGA
**miR-30c-1**	miR-30c-5p	UGUAAACAUCCUACACUCUCAGC	miR-30c-1-3p	CUGGGAGAGGGUUGUUUACUCC
**miR-186**	miR-186-5p	CAAAGAAUUCUCCUUUUGGGCU	miR-186-3p	GCCCAAAGGUGAAUUUUUUGGG
**miR-199b**	miR-199b-3p	ACAGUAGUCUGCACAUUGGUUA	miR-199b-5p	CCCAGUGUUUAGACUAUCUGUUC
**miR-200b**	miR-200b-3p	UAAUACUGCCUGGUAAUGAUGA	miR-200b-5p	CAUCUUACUGGGCAGCAUUGGA
**miR-330**	miR-330-5p	UCUCUGGGCCUGUGUCUUAGGC	miR-330-3p	GCAAAGCACACGGCCUGCAGAGA
**miR-335**	miR-335-5p	UCAAGAGCAAUAACGAAAAAUG	miR-335-3p	UUUUUCAUUAUUGCUCCUGACC
**miR-346**	miR-346-5p	UGUCUGCCCGCAUGCCUGCCUCU	miR-3460-3p	AGGCAGGGGCUGGGCCUGCAGC
**miR-466**	miR-466-5p	AUACACAUACACGCAACACACAU	miR-465-3p	GUGUGUUGCAUGUGUGUAUAUGU
**miR-574**	miR-574-5p	UGAGUGUGUGUGUGUGAGUGUGU	miR-574-3p	CACGCUCAUGCACACACCCACA
**miR-3126**	miR-3126-5p	UGAGGGACAGAUGCCAGAAGCA	miR-3126-3p	CAUCUGGCAUCCGUCACACAGA
**miDNA**	miDNA	taatactgcctggtaatgatga	miDNA *	catcttactgggcagcattgga

* Upper character indicates RNA. Lower character, DNA. s* indicates miRNA strand which is not assigned in miRBase.

Table S2. Oligonucleotide sequences for construction of miRNA target reporters. Each oligonucleotide contains completely matched target sequence of each miRNA (22 nt) with 2 nt excessive sequences (upper cases) at both ends, and the overhang sequence of restriction enzyme (4 nt, lower cases) at 5' end.

	Sense strand	Antisense strand
**let-7b-5p target**	tcgaGAAACCACACAACCTACTACCTcacc	aattGGTGAGGTAGTAGGTTGTGTGGtttc
**miR-21-5p target**	tcgaAGTCAACATCAGTCTGATAAGCtacc	aattGGTAGCTTATCAGACTGATGTTgact
**miR-200b-3p target**	tcgaCGTCATCATTACCAGGCAGTATtaga	aattTCTAATACTGCCTGGTAATGATgacg
**miR-330-5p target**	tcgaGAGCCTAAGACACAGGCCCAGAgagg	aattCCTCTCTGGGCCTGTGTCTTAGgctc

Table S3. qRT-PCR was carried out using the Forward and the Reverse primers.

Gene name	Forward primer (5'→3')	Reverse primer (5'→3')
**HDHD1A**	GACGGACTTCTTCTGGATACTG	CCATAACCAGGGACTTTACATC
**UBQLN1**	CAGCTACAGAATCCAGAAGTCAG	CCCAGTAACCTTTCAATAGCTG
**PBX2**	GGAGGCAAACATCTATGCTG	CCAGATCCTGAGAGATTGAAAG
**ZC3H11A**	CAGTCCTCTTCAGATTCCTCAC	AAATCATCCTCCACAGAGAGTG
**SKA2**	CCAGGTTCAAGCAGTTCTCTG	GGATCACCATCAGGAGTTAAAG
**CASP7**	ATCAGGGCTGTATTGAAGAGC	TACTGAAGAGGGACGGTACAAAC
**SLC11A2**	CTTGGTCCTTATCATCTGTTCC	CAACCCAAGTAGAACACAAAGC
**UBR3**	CGACTAAATAGCTGTCCTTTCC	ACCACACTTACAAATGGTCTGG
**RARG**	ATCAGCACTAAGGGAGCTGAAAG	CTCAAACATTTCAGGGTTCTCC
**SPAST**	GTGAAGAATATGTCTGCCAGTG	CTTCTAAAGTTTGAGGGCTGAC
**METTL7A**	CTATTTCATGGAGCATGTGG	AGCTCTCTCTGGTCAGGTTG
**SEC23A**	CGGTTCTTCTTGATAGCAGTAGC	CATATCCTGGTATCCTGACTTCC
**HMGN4**	GCTCGGTTGTCTGCTAAACC	CTTTCCAGCATCTGCTTTCC
**ZNF652**	GAGTAAGGAAATGCTTCTCACC	GTAACTGACAGAGAGGGAGCAC
**SLC39A1**	GTCACCTCTGGAGGAAACAAG	CAGGGAGAACACCAGTACACAG
**OTUD4**	TTCAGCCATCCTAGAAACTAGC	ACACTAGAGTGGACCTTGCTTAG
**USP37**	CCTAACACCAGATTCAAGTCAC	CTTGCAAGAGTGCATTACTCAG
**AP1S2**	TGAGCACTGTCTTATCACATCG	CAGCAGCTCTGCATAGTTTGAC
**OSTM1**	TCAAGAGATCCTCCTGCCTTAG	CCAACTAGACTCTGGGCTACAC
**SHCBP1**	CAAGGAAGGGATCCTCATTAAG	TCACCAAGACAACACCATAACC
**PGM2L1**	CTACCTTCCAAGTGAGTCACAAC	CCTCACCATACATAGAAGCAGAG
**TOMM34**	TCAGAAGAGGTGGAATTCCTTG	TTGCCTTCTTCCTTCAGAACTC
**SPRYD3**	GTCTGTGGCTTATCATGCAGAC	ATTCCACAGCCCATGATGTC
**SRPR**	TCAAAGAGCTTGAGTCGTGAAG	CAACAGATTCACAGAGCTGGAC
**B4GALT1**	GGGAGTTGTGGGAAATAAGAAG	AAGTTACCACTCAGGGCATTTG
**SLC7A11**	GCATCTGCTACCTTTGAGAGTG	CTGCCCATACCAGAAAGCTC
**NHLRC3**	CAGGTTTACTCCTGATGGGAAG	TGATCTGCTAGTTGGATTGTGC
**CLDN12**	GTACTCCCATCCACCCAGTATG	CCCATTAAGTGGTGTGTGAAAC

© 2013 by the authors; licensee MDPI, Basel, Switzerland. This article is an open access article distributed under the terms and conditions of the Creative Commons Attribution license (http://creativecommons.org/licenses/by/3.0/).
